# Basis function compression for field probe monitoring

**DOI:** 10.1002/mrm.30471

**Published:** 2025-02-18

**Authors:** Paul I. Dubovan, Gabriel Varela‐Mattatall, Eric S. Michael, Franciszek Hennel, Ravi S. Menon, Klaas P. Pruessmann, Adam B. Kerr, Corey A. Baron

**Affiliations:** ^1^ Department of Medical Biophysics Western University London Ontario Canada; ^2^ Centre for Functional and Metabolic Mapping Western University London Ontario Canada; ^3^ Lawson Health Research Institute London Ontario Canada; ^4^ Institute for Biomedical Engineering ETH Zurich and University of Zurich Zurich Switzerland; ^5^ Center for Cognitive and Neurobiological Imaging Stanford University Stanford California USA; ^6^ Department of Electrical Engineering Stanford University Stanford California USA

**Keywords:** basis functions, diffusion MRI, eddy currents, expanded encoding, field monitoring, spherical harmonics

## Abstract

**Purpose:**

Field monitoring using field probes allows for accurate measurement of magnetic field perturbations, such as from eddy currents, during MRI scanning. However, errors may result when the spatial variation of the fields is not well‐described by the conventionally used spherical harmonics model that has the maximum order constrained by the number of probes. The objective of this work was to develop and validate a field monitoring approach that compresses higher order spherical harmonics into a smaller set of new basis functions that can be characterized using fewer probes.

**Methods:**

Field monitoring of acquisitions was repeated with probes in different locations. High‐order field dynamics were computed from this “calibration” data assembled from provided scans, from which compression matrices could be devised using principal component analysis. Compression matrices were then used to fit field dynamics using “compressed” basis functions with data from 16 probes, which were then used in image reconstruction. Performance was evaluated by assessing the accuracy of computed field dynamics as well as in vivo image quality. Technique generalizability was also assessed by using various acquisition and diffusion encoding strategies in the calibration.

**Results:**

Qualitative and quantitative improvements in accuracy were observed when using the proposed fitting method compared to the conventional approach. However, compression effectiveness was influenced by the probe quantity and arrangement, and the specific acquisition data included in the calibration.

**Conclusion:**

The ability to tailor basis functions to more compactly describe the spatial variation of field perturbations enables improved characterization of fields with rapid spatial variations.

## 1 INTRODUCTION

Field monitoring is an effective tool for measuring field perturbations during acquisitions because of its high sensitivity to spatial and temporal field variations.^1^, ^2^, ^3^, ^4^ This technique enables the accurate measurement of spatially invariant field offsets, first‐order gradients, and higher order field deviations arising primarily from gradient‐induced eddy currents.^5^, ^6^ Of note are eddy currents generated by the strong diffusion gradients used in diffusion encoding sequences. These include pulsed gradient spin echo (PGSE),^7^ oscillating gradient spin echo (OGSE),^8^, ^9^, ^10^ and spherical tensor encoding schemes,^11^, ^12^ which are notorious for introducing large field deviations.^13^ When unaccounted for, these fields can produce notable image artifacts and errors in computed diffusion metrics such as mean diffusivity and fractional anisotropy (FA),^14^ as well as in advanced parameters like kurtosis^15^ and microscopic fractional anisotropy (*μ*FA).^16^, ^17^, ^18^ To account for these effects, field monitoring measurements can be incorporated in image reconstruction strategies, thereby reducing artifacts and improving the accuracy of associated diffusion metrics.^19^, ^20^

The most widely used commercial field monitoring system (Skope) uses 16 field probes that record the local MR signal phase, which allows computation of the local magnetic field. This allows for characterization of spatial variations using real‐valued spherical harmonics up to 16 basis functions, corresponding to third order.^1^ For most field monitoring uses, this is not problematic as fitting to second or third order is sufficient to capture most field perturbations. However, in some cases phase contributions originating from higher orders may be non‐negligible, such as when using head‐only MRI scanners or high‐performance gradient systems that operate at very high gradient strengths and slew rates.^21^, ^22^, ^23^ In addition, scenarios may exist where field probes are located in regions that experience rapidly varying eddy current modes and gradient nonlinearity, which may not be well characterized by spherical harmonics fit using only 16 probes.^24^, ^25^ This can lead to biased or erroneous fitting of the phase amongst the available spatial orders, including the lower orders that best describe the field variations most commonly experienced in the imaging region, and in turn corrupted image quality. To overcome these challenges, fitting the basis functions using more field probes than functions leads to an overdetermined system, and hence, a better conditioned least‐squares problem that reduces the error in calculated coefficients.^1^ Integrating more probes also permits the characterization of higher spatial orders, which may help to more accurately fit the probe phase if higher orders are present. Although the number of probes is limited by hardware and available space, the generation of additional field probe measurements can be accomplished by repeating the acquisition after moving the probes into different positions, from which the probe data from the separate scans can be compiled together to form a larger probe array. Although advantageous, this approach is time consuming and not possible for concurrent field monitoring given the subject's presence in the coil.

In this work, we propose and test a “basis function compression” strategy to accurately calculate high‐order field dynamics from a limited number of field probes. Using a head‐only 7 T MRI, we demonstrate greatly improved reconstruction quality for a transmit‐receive coil with 16 integrated field probes where all probes extend into the nonlinear region of the gradient.

## 2 METHODS

The compression technique has been made publicly available (see Data Availability Statement).

### 2.1 Determination of compressed k‐coefficients

#### 2.1.1 Establishing ground truth calibration k‐coefficients

The phase accrued by field probes during an acquisition can be described as separable in space and time and can be expressed as the spherical harmonics evaluated at the spatial positions of the probes multiplied by the “k‐coefficients” that describe the time‐dependence of each basis function. For more details on the derivation of phase coefficients using field probes, please refer to the author's previous work,^24^ and the sections described in Barmet et al.^1^ and Wilm et al.^19^ Here, we consider a “calibration scan” that consists of multiple acquisitions with various gradients applied to excite a variety of eddy current modes, such that a total of $N_{t}$ samples are acquired across all time points and gradient waveforms. To increase the total number of field probe positions, this scan is repeated multiple times with probes in different locations, arriving at a total of $N_{p}$ probe locations. The phase accrual of the probes is given by:

(1) $$\phi_{\text{calib}}=P_{\text{calib}}k_{\text{calib},}$$

where $\phi_{\text{calib}}^{N_{p}\times N_{t}}$ is the phase, $P_{\text{calib}}^{N_{p}\times N_{b}}$ is the “probing matrix” that contains $N_{b}$ solid harmonic basis function values at the location of each probe, and $k_{\text{calib}}^{N_{b}\times N_{t}}$ are the k‐coefficients that correspond to each basis function. Accordingly, $k_{\text{calib}}$ can be determined from the calibration data $\phi_{\text{calib}}$ using a Moore‐Penrose pseudoinverse of $P_{\text{calib}}$.

#### 2.1.2 Basis function compression

The goal of basis function compression is to more compactly represent the typical field perturbations experienced on a system compared to the solid harmonic basis functions that are typically used. Similar to Wilm et al.,^21^ a set of compressed k‐coefficients can be determined as weighted combinations of solid harmonic k‐coefficients using an economic singular value decomposition (SVD) of the ground truth harmonic coefficients determined from the calibration scan. Because typical gradient systems are designed to generate linearly varying phase distributions, it is reasonable to assume a priori that the zeroth and first order solid harmonics are effective basis functions that can, therefore, be omitted from the SVD:

(2) $$\Gamma^{\text{high}}k_{\text{calib}}^{\text{high}}=U\sum V^{T},$$

where the “high” superscript denotes that only second and higher order terms are included. $\Gamma^{\text{high}}$ is a diagonal matrix of weights that reduces bias in the SVD from signal and noise amplification that can occur from the different scalings of each order. In this work, we set $\Gamma_{i,i}^{\text{high}}={\left(0.1\right)}^{l_{i}}$, where $l_{i}$ is the order of the *i*'‐th basis function, having *i* = 1 start at the second order basis functions. This choice of Γ is equivalent to weighting the basis functions the same over a standard imaging volume with a 10‐cm radius, or to converting the units of k‐coefficients from rad/m^*l*^ to rad/dm^*l*^.

The singular vectors in U describe linear combinations of k‐coefficients that most compactly describe the calibration data. Therefore, a compression matrix can be defined by omitting the columns corresponding to the lowest singular values, similar to algorithms for coil compression:^26^, ^27^

(3) $$C^{\text{high}}\equiv U_{i,j},0<i<\left(N_{b}-4\right),0<j<L,$$

where the “high” subscript denotes this compression matrix corresponds to the harmonic orders of 2 and higher, *L* is the number of singular vectors retained (which may be based on singular value thresholding), and 4 is subtracted from $N_{b}$ because the zeroth and first order terms were omitted in Eq. (2). To create weighting and compression matrices that can be applied to all orders, block diagonal matrices can be constructed as follows:

(4) $$\Gamma =\left[\begin{array}{ll} I & 0\\ 0 & \Gamma^{\text{high}} \end{array}\right]\;\text{and}\;C=\left[\begin{array}{ll} I & 0\\ 0 & C^{\text{high}} \end{array}\right],$$

where I is an 4×4 identity matrix that retains the zeroth and first order k‐coefficients. Accordingly, k‐coefficients for an arbitrary scan can be compressed via:

(5) $$\hat{k}=C^{T}\Gamma k.$$

Likewise compressed k‐coefficients can be uncompressed via:

(6) $$k\approx \Gamma^{-1}C\hat{k},$$

where the equality is approximate because of the discarding of some singular vectors. Compared to the full set of k‐coefficients, there are a smaller number of compressed k‐coefficients and, therefore, fewer probes would be needed to compute them. For routine image reconstruction using field monitoring measurements from a conventional 16‐probe array, a maximum of 12 higher order compressed basis functions is permitted, given that 4 basis functions are allocated to the uncompressed zeroth and first order terms. Substituting Eq. (6) into Eq. (1) to recast Eq. (1) into a form with compressed k‐coefficients yields:

(7) $$\phi =P\Gamma^{-1}C\hat{k,}$$

where ***P*** and $\hat{k}$ represent the probing matrix and compressed k‐coefficients, respectively. Equation (7) can be observed to have a similar format as Eq. (1) with the substitution:

(8) $$\hat{P}\equiv P\Gamma^{-1}C,$$

which leads to $\phi =\hat{P}\;\hat{k}$. Here, $\hat{P}$ can be interpreted as representing a compressed set of basis functions that correspond to the compressed k‐coefficients. Accordingly, the compressed k‐coefficients can be determined from the phase data using the pseudoinverse of $\hat{P}$:

(9) $$\hat{k}={\left({\hat{P}}^{T}\hat{P}\right)}^{-1}{\hat{P}}^{T}\phi .$$

**FIGURE 1:**
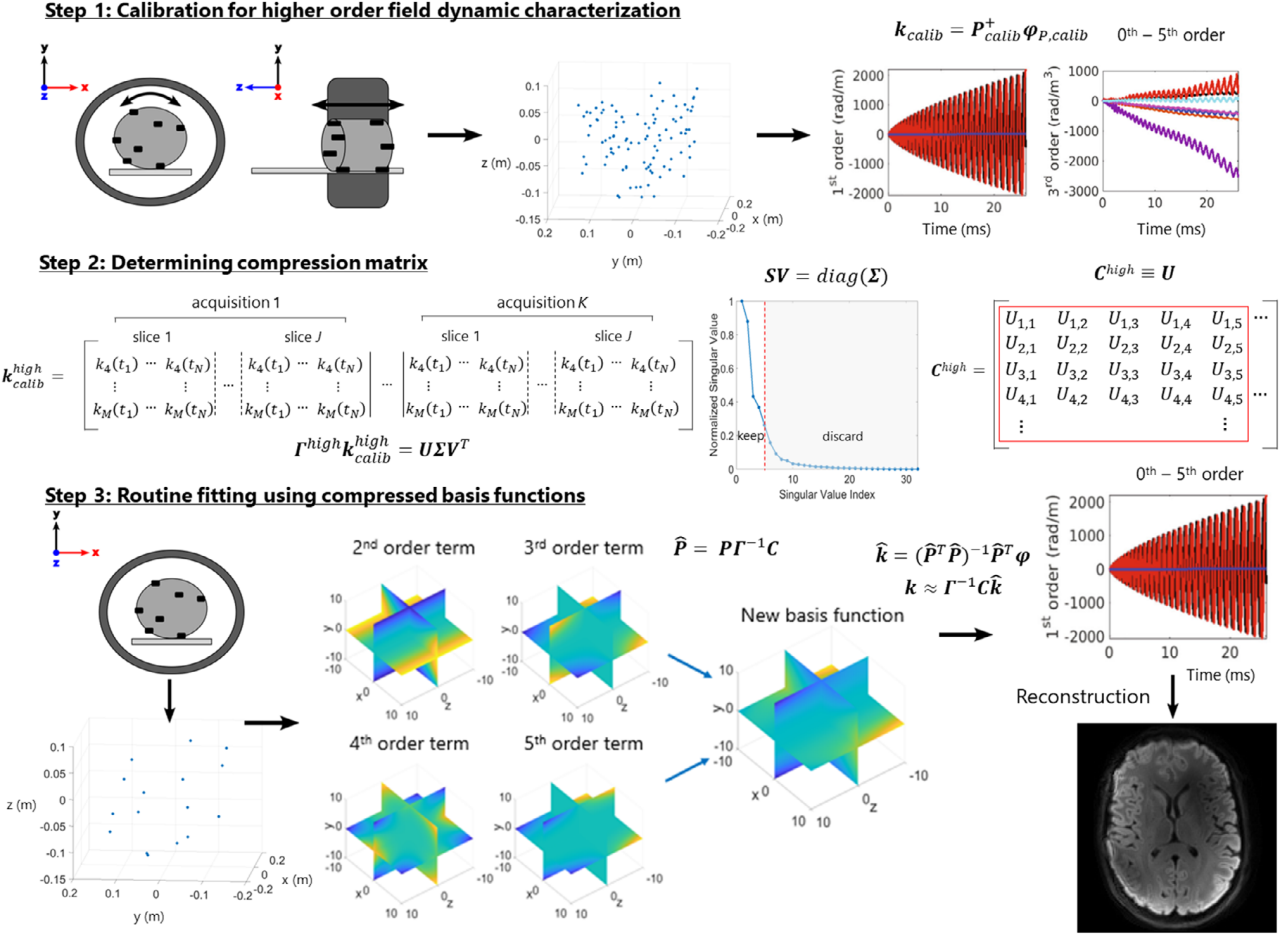
An outline of the typical compression process. In the first step, higher order field dynamics are characterized using a compilation of probe measurements acquired using various probe positions. Principal component analysis is then performed on the desired higher order phase coefficient time‐courses, from which a compression matrix is determined in step 2. The matrix is truncated based on inspection to preserve only the most relevant basis functions. Last, the compression matrix can be applied to compress basis functions sampled using the conventional probe arrangement, from which decompressed higher order field dynamics can be retrieved for use in an expanded encoding model‐based reconstruction.

After computing $\hat{k}$ from Eq. (9), Eq. (6) can be used to retrieve the k‐coefficients in their original uncompressed solid harmonic form, which can then be used in existing expanded encoding reconstruction pipelines that use these basis functions. A detailed diagram of the process is illustrated in Figure 1.

### 2.2 Scan details

Scanning was conducted on a 7 T head‐only MRI scanner (Siemens MAGNETOM Terra Plus) equipped with an AC‐84II head gradient coil (80 mT/m max gradient and 400 T/m/s max slew rate) at Western University's Centre for Functional and Metabolic Mapping. This study was approved by the institutional review board, and informed consent was obtained before scanning. Field monitoring was performed using a commercial Clip‐On Camera (Skope) consisting of 16 probes integrated into a 32‐channel receive, 8‐channel transmit RF head coil.^25^

#### 2.2.1 Calibration acquisition details

Field monitoring measurements from different probe locations were performed by rotating the field probe coil about the z‐axis and translating the coil along the z‐direction, where identical acquisitions were performed in each location with no imaging subject. Nine different coil orientations were used: 3 z‐positions (spaced ˜4–5 cm from each other), and three equiangular rotated positions for each given z‐location. From this collection of probe positions, a synthetic probe array consisting of 100 probes was generated using probes positioned up to a maximum Euclidean distance of 16 cm from isocenter, as well as within individual x, y, z distances of 14 cm from isocenter to avoid excessive field inhomogeneity and gradient nonlinearity. For this experiment, fifth order fits were performed using the calibration data with Eq. (1) to establish ground truth k‐coefficients. Unless stated otherwise, $k_{\text{calib}}$ included data from all acquisition time points, slices, b_0_, and diffusion weighting volumes.

The success of compression depends on the ability of the calibration data to accurately represent all the field perturbations that might be encountered in scans that the compression matrix is applied to. Assuming that the dominant source of perturbations is gradient‐induced eddy currents, the calibration data should include various gradient waveforms. On one extreme, calibration data could contain nearly all possible gradient waveforms, such as a collection of chirped or triangular pulses typically used to calibrate gradient impulse response functions.^28^, ^29^ Alternatively, one could use calibration data that is very similar to the desired acquisitions, such as a collection of spiral acquisitions at various resolutions to calibrate for spiral acquisitions only. The former approach has the advantage of generalizability, but it may not be very compressible compared to the latter. To explore these trade‐offs, various calibration scans were performed (Table 1). Notably, any number of these calibration scans can be combined to obtain a single compression matrix from Eqs. ((1), (2), (3), (4))–((1), (2), (3), (4)), where combining different scans is expected to improve generalizability at the expense of compressibility. All DTI acquisitions consisted of 2 *b* = 0 s/mm^2^ acquisitions and 30 diffusion directions, which were uniformly distributed using electrostatic repulsion of particles on a sphere.^30^ Other common imaging parameters include FOV: 192 × 192 mm^2^, slice thickness: 2 mm, number of slices: 10, axial orientation, rate 2 undersampling, scan time approximately 1.5 min for each coil location. Differing scan parameters are described in Table 1.

**TABLE 1:** Calibration acquisition details.

Scan description	Resolution	TE	TR	Bandwidth	Diffusion weighting
*Scan 1*	1.3 × 1.3 × 2 mm^2^	39 ms	2500 ms	2014 Hz/Px	PGSE encoding
Higher res spiral PGSE					*b* = 1000 s/mm^2^
*Scan 2*	2 × 2 × 2 mm^2^	39 ms	2500 ms	4006 Hz/Px	PGSE encoding
Lower res spiral PGSE					*b* = 1000 s/mm^2^
*Scan 3*	1.3 × 1.3 × 2 mm^2^	93 ms	2500 ms	2014 Hz/Px	OGSE encoding
Spiral OGSE					*b* = 400 s/mm^2^
					frequency = 40 Hz
*Scan 4*	1.3 × 1.3 × 2 mm^2^	53 ms	2500 ms	2140 Hz/Px	PGSE encoding
EPI PGSE					*b* = 1000 s/mm^2^

*Note*: To assess the impact of readout gradient frequency content on basis function compression, identical single‐shot spiral diffusion‐weighted acquisitions with different in‐plane resolutions and resulting bandwidths were performed. Additionally, an OGSE scheme using 40 Hz oscillating diffusion gradients^10^, ^46^ was conducted to explore how frequency content of diffusion gradients may affect compression. Last, an EPI scan with similar imaging parameters as the higher resolution spiral acquisition was performed to explore the compression performance when calibrating with a different trajectory.

Abbreviations: OGSE, oscillating gradient spin echo; PGSE, pulsed gradient spin echo.

#### 2.2.2 In vivo imaging

One healthy volunteer was scanned with scans 1 to 4, and field monitoring was performed concurrently.^31^ In this baseline position, the mean distance between field probes and isocenter is 13.5 cm (range: 11.9–14.9 cm), with all probes being 8% to 35% outside the 22 cm diameter spherical volume (DSV). A second healthy volunteer was scanned using a single‐shot spiral b‐tensor encoding acquisition^11^, ^12^ with concurrent field monitoring. The following imaging parameters were used: FOV: 200 × 200 mm^2^, 1.5 mm isotropic resolution, 88 slices, TE/TR: 82/10500 ms, undersampling rate 3, bandwidth = 2194 Hz/Px. The protocol consisted of a 94‐direction diffusion scheme with 6 *b* = 0 s/mm^2^ volumes, 6, 26, and 26 linear tensor‐encoded (LTE) volumes at *b* = 150, 1000, and 2000 s/mm^2^, respectively, and 30 spherical tensor‐encoded (STE) volumes at *b* = 2000 s/mm^2^, totaling a scan time of 16.5 min.

For all in vivo scans, Cartesian dual‐echo gradient‐echo acquisitions were used to estimate B_0_ maps for inclusion in a model‐based reconstruction to correct for static off‐resonance effects. The imaging parameters were as follows: FOV = 210 × 210 mm^2^, 2 mm isotropic resolution, 74 slices, TE_1_/TE_2_ = 4.08/5.10 ms, TR = 542 ms.

### 2.3 Image reconstruction

Image reconstruction was performed using an iterative expanded encoding model‐based reconstruction^19^ in MATLAB via the MatMRI toolbox.^32^ All images were reconstructed using the conjugate gradient method with Tikhonov regularization and a regularization weighting of 0.2. All reconstructions converged within 10 to 30 iterations. Coil compression to 24 virtual coils was performed to improve reconstruction speed.^26^, ^27^, ^33^, ^34^ ESPIRiT was used to estimate sensitivity coil maps.^35^ Synchronization delay between the MRI and field probe data was corrected using an automatic, retrospective algorithm.^36^ Noise correlation between receivers was corrected using prewhitening before any reconstructions.^37^ Field dynamics were also adjusted to account for vendor Maxwell corrections and eddy current compensation that are measured by the field probes, using methods described previously by the authors.^25^ Unless stated that “uncorrected” k‐coefficients were used, all k‐coefficient fitting performed for data acquired on our scanner, even when implementing conventional fits, included a weighting function *W* that suppresses errors from distal field probes, as described in previous work.^24^ Accordingly, *P* is replaced by *WP* in the equations, producing ϕ=WPk and $k={\left(P^{T}W^{2}P\right)}^{-1}P^{T}W^{2}\phi$ for k‐coefficient calculation (likewise for $\hat{P}$). Similar to previous work,^24^ concomitant fields were included in all k‐coefficient fitting, including compressed fitting, by iterative computation of concomitant gradient phase from the linear k‐coefficients after evaluating Eq. (9), removal of concomitant phase from ϕ, followed by re‐evaluation of Eq. (9).

Following image reconstruction, FA maps of in vivo images were computed using the MRtrix3 package.^38^ Additionally, b‐tensor encoding volumes were denoised by performing principal component analysis denoising on the complex data,^39^ and ADC and μFA maps were calculated using matMRI.^40^

### 2.4 Data analysis

To quantitatively assess basis function compression performance, k‐coefficient root‐mean‐square‐error (RMSE) was calculated from the concurrently monitored acquisitions with 16 probes with respect to the ground truth k‐coefficients, $k_{\mathrm{GT}}$, computed from the full 100 probe locations from the calibration scans. Earlier work has shown that inadequate modeling of the spatial variation of field dynamics causes the omitted higher orders to be projected to the lower orders, and that it is errors in the lower order k‐coefficients that are primarily responsible for image artifacts.^24^ Accordingly, RMSE was only computed for the first nine basis functions, corresponding to the zeroth to second order, unless otherwise stated. For similar rationale as in Section 2.1.2, $\Gamma_{i,i}={\left(0.1\right)}^{l_{i}}$ was applied as well, except here the basis functions start at the zeroth order:

(10) $$\text{RMSE}=\sum_{i=1}^{9}\sqrt{\frac{1}{N_{t}}\sum_{j=1}^{N_{t}}{\left(\Gamma \left(k_{i,j}-k_{i,j}^{\mathrm{GT}}\right)\right)}^{2}},$$

The RMSE was computed separately for each volume (slices, b_0_ and diffusion acquisitions, unless otherwise stated), followed by calculation of the mean and SD across all volumes. To quantitatively assess the performance of the technique in terms of image quality, DWI and FA map errors were assessed relative to ground truth images (i.e., images reconstructed with ground truth k‐coefficients) using normalized‐root‐mean‐squared‐error (NRMSE) and structural similarity index (SSIM), followed by calculation of the mean and SD across all volumes. Statistical comparisons of these error metrics when comparing the different fitting strategies (conventional vs. compressed) were performed using paired *t* test analysis (α = 0.05) across all included volumes. Qualitative assessments were performed from image reconstructions and, similar to Wilm et al.,^21^ for phase maps from compressed basis functions in planes orthogonal to two cardinal axes.

### 2.5 Technique performance: Additional sites

To assess the performance of the technique on different infrastructure, field monitoring data was collected from ETH Zurich (3 T Philips Achieva scanner, 200 mT/m max gradient and 600 T/m/s max slew rate, ˜20‐cm imaging volume^41^) and Stanford (GE 3 T UHP scanner, 100 mT/m max gradient, 200 T/m/s max slew rate, 50‐cm imaging volume). The ETH Zurich acquisition implemented a single‐shot spiral diffusion‐weighted acquisition like the Western University data and consisted of both in vivo and field monitoring measurements, although here, field monitoring was acquired during a separate acquisition. The Stanford data consisted of an acquisition of chirped gradient pulses and only considered field monitoring measurements. Both setups used a commercial Dynamic Field Camera (Skope) with optimized probe placements. Specific details are described in Data S1.

## 3 RESULTS

Performing reconstructions informed by third and fifth order fits calculated using the 100‐probe array showed substantial improvements in DWI and FA maps over reconstructions informed by third order fits calculated conventionally using the 16‐probe arrangement. Incremental improvements were observed when comparing fifth to third order fit with 100 probes (Video S1), suggesting that fourth and fifth order terms have little impact on image reconstruction provided that the lower order k‐coefficients are accurate.

**FIGURE 2:**
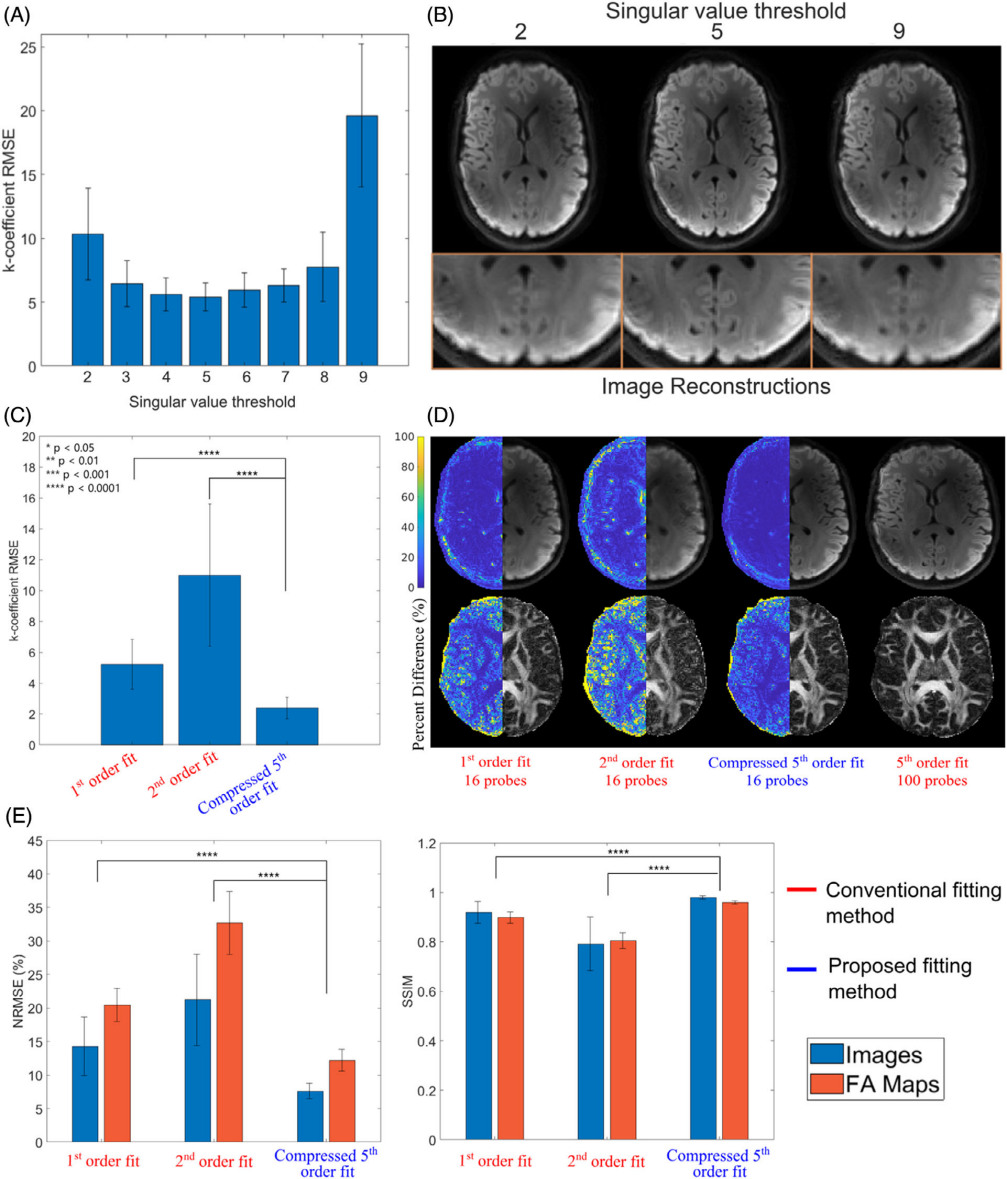
Investigation of singular value threshold compression performance for scan 1. Shown in (A) is the average root‐mean‐square‐error (RMSE) of up‐to‐second‐order computed k‐coefficients relative to the ground truth as a function of the singular value quantity preserved for compression. (B) Presents the reconstructed mean DWI informed by the compressed k‐coefficients using the outlined number of singular values. Zoom‐ins highlight the substantial blurring experienced at the low and high singular value regimes. (C) Illustrates the error for first‐order k‐coefficients relative to the ground truth (fifth order fit using 100 probes) for conventional first and second order fits and compressed fifth order fit using five singular values for compression. (D) Respective reconstructed mean DWI and calculated fractional anisotropy (FA) maps when incorporating the same fitting schemes. Percent difference images were calculated relative to images informed by fifth order field dynamics and are shown in the left hemisphere of the images. (E) Respective normalized‐root‐mean‐squared‐error (NRMSE) and structural similarity index (SSIM) comparisons of reconstructed image volumes and FA maps relative to the ground truth images. Comparisons with conventional third order fits are not shown because of the absence of an overdetermined state and evidently higher errors exhibited (Video S1).

When truncating the compression matrix to different numbers of singular vectors when using scan 1 for both calibration (100 probes) and k‐coefficient calculation from probe measurements during the in vivo scan (16 probes), the RMSE plot and resulting images showed the lowest RMSE and best image quality when using five singular values (Figures 2A,B and S1). Using five singular values, computed compressed fifth order‐fit k‐coefficient plots exhibited the lowest RMSE (Figure 2C), showing better similarity for first order terms when compared with conventional first and second order fits (Figure S2). Mean DWI and FA maps reconstructed from a compressed fifth order fit were of comparable quality to images reconstructed from ground truth k‐coefficients computed from the scan 1 calibration data, whereas image quality was significantly degraded when using conventional fitting schemes, especially when increasing the fitting order (Figure 2D). Quantitative image analysis further supported these trends as NRMSE and SSIM for the image volumes, and FA maps were, respectively, the lowest and highest by a significant margin when using the compressed fit with SSIM values close to 1 being achieved (Figure 2E). The total number of terms used in the compressed fit (uncompressed lower order and compressed higher order) equaled nine, which is equivalent to the total number of terms included in a second order fit, yet the compressed fifth order fit performed markedly better.

**FIGURE 3:**
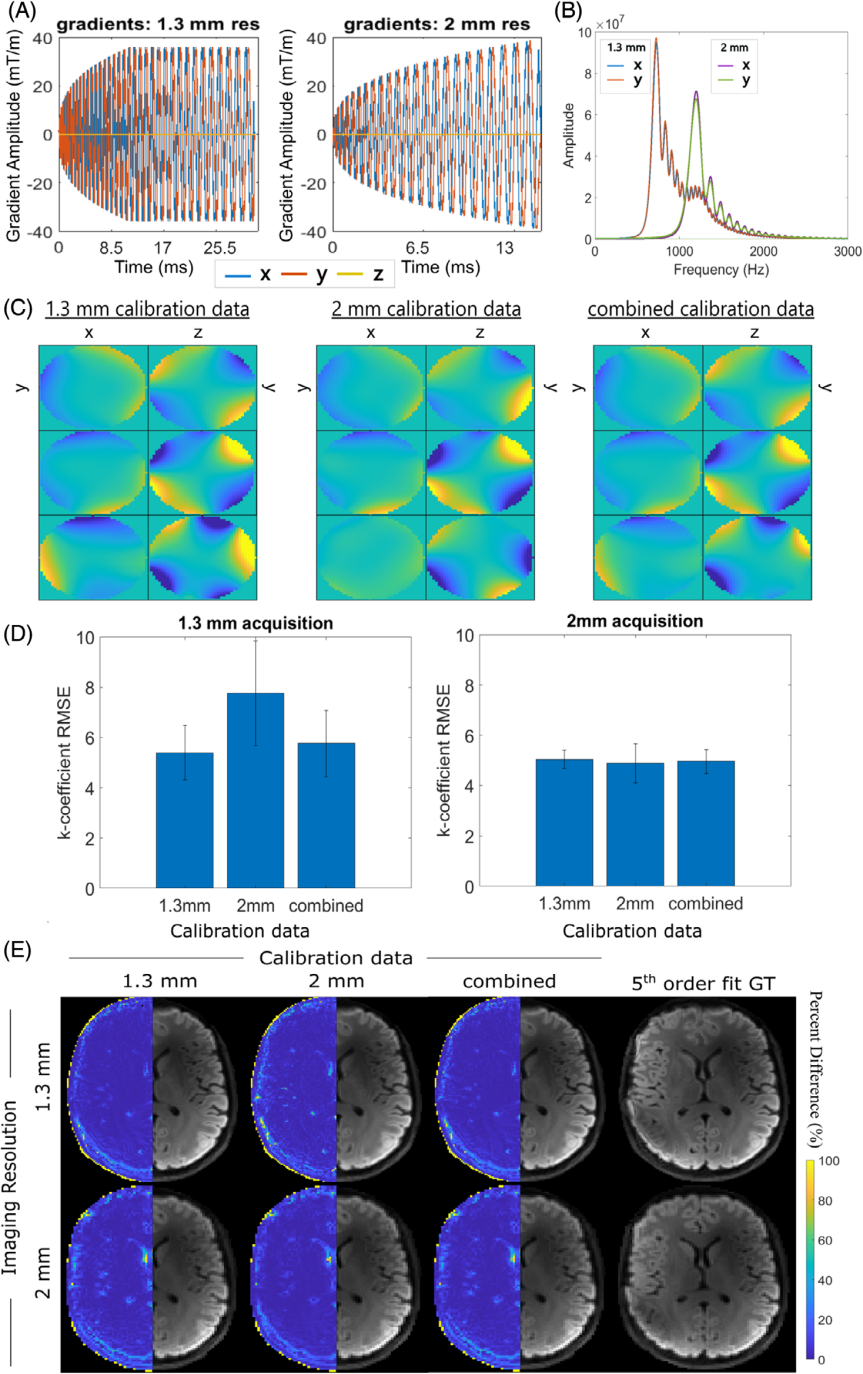
(A) Gradient readout profiles for the 1.3‐ and 2‐mm in‐plane acquisitions, and (B) respective power spectral density profiles. (C) Cross‐sectional spatial distribution (left‐to‐right: x‐y, y‐z planes) of compressed basis functions when using low, high, and combined resolutions for the calibration data. Rows represent the compressed basis functions related to the first three principal components. (D) Mean root‐mean‐square‐error (RMSE) analysis of up‐to‐second‐order k‐coefficients, using low, high, and combined resolutions for the calibration data. Comparison was performed for field dynamics measured for the 1.3‐mm acquisition (left) and 2‐mm acquisition (right). (E) Mean DWI reconstructions for the 1.3‐mm (top) and 2‐mm (bottom) acquisitions, when informed by field dynamics compressed based on the described calibration data: left‐to‐right 1.3‐mm acquisition, 2‐mm acquisition, combined resolutions, plus respective fifth order fits calculated using 100 probes. Percent difference images were calculated relative to images informed by fifth order field dynamics and are shown in the left hemisphere of the images. Five singular values were kept for compression.

The gradient profiles for the spiral trajectories acquired with different resolutions are illustrated in Figure 3A. Frequency content analysis showed significant separation of power spectral density peaks for the *x* and *y* gradient channels, with overlap in the overall spectra also being apparent (Figure 3B). The combination of both resolutions into the compression matrix resulted in maps most comparable to the higher resolution case (Figure 3C). Both resolutions had the lowest RMSE when the same scan was used to generate calibration data, but the combined calibration data performed well for both resolutions. The RMSE was not very sensitive to choice of calibration data for the 2‐mm resolution scan (Figure 3D), likely because of the gradient spectra of the 1.3‐mm resolution scan having high overlap with the whole 2‐mm resolution scan. Little qualitative differences in DWI were seen at either resolution, regardless of the calibration data used for compression (Figure 3E).

**FIGURE 4:**
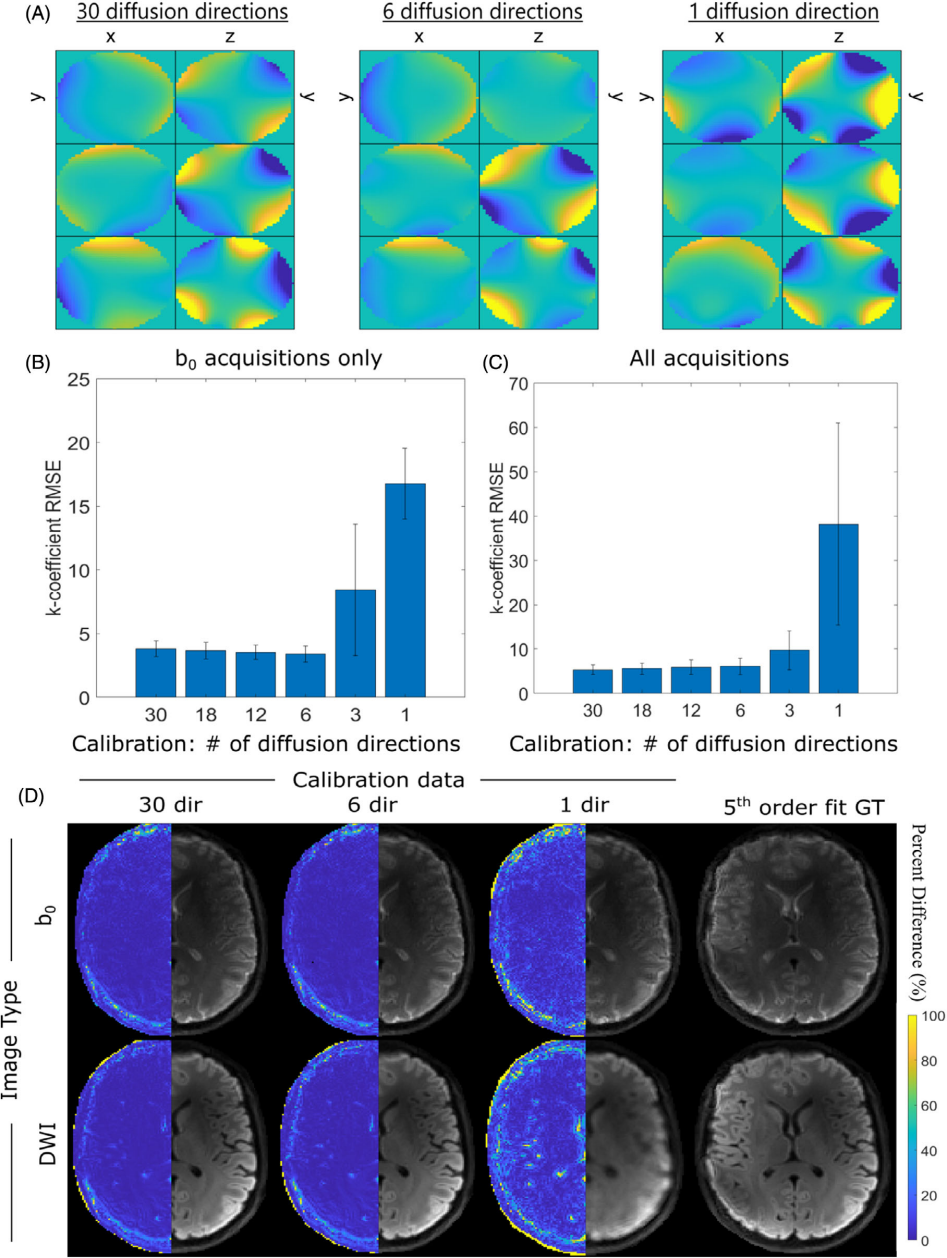
(A) Cross‐sectional spatial distribution (left‐to‐right: x‐y, y‐z planes) of compressed basis functions for calibration data equipped only with diffusion data containing 30, 6, 1 directions. b_0_ Acquisitions were omitted from the calibration data itself for all relevant analysis. Rows represent the new basis functions related to the first three principal components. Mean root‐mean‐square‐error (RMSE) analysis of up‐to‐second‐order k‐coefficients, when preserving the following amount of uniform diffusion directions in calibration: 30, 18, 12, 6, 3, 1, for (B) b_0_ acquisitions only, and (C) when including the diffusion acquisitions in the analysis. (D) Mean b_0_ (top) and DWI (bottom) reconstructions when informed by field dynamics compressed using (left‐to‐right) 30, 6, 1 diffusion directions in the calibration data, plus respective fifth order fits calculated using 100 probes. Percent difference images were calculated relative to images informed by fifth order field dynamics and are shown in the left hemisphere of the images. Five singular values were kept for compression. Directions in the subsets were chosen to maximize electrostatic repulsion for each subset.

Figure 4A shows that strong similarity in basis function maps was maintained as the number of diffusion directions was reduced from 30 to six, but a complete mismatch was observed when only one diffusion direction was included in the calibration. RMSE analysis was performed for diffusion‐calibration data that incorporated one to 30 diffusion directions and was assessed for b_0_‐only acquisitions (Figure 4B), as well as for all acquisitions (Figure 4C). For this specific analysis, calibration data did not include b_0_ acquisitions given that the assessment of diffusion direction quantity was of interest. In both cases, RMSE increase was only observed below six directions. Similarly, image quality was similar for calibrations using 30 and six directions, which drastically lowered when using a single direction (Figure 4D).

**FIGURE 5:**
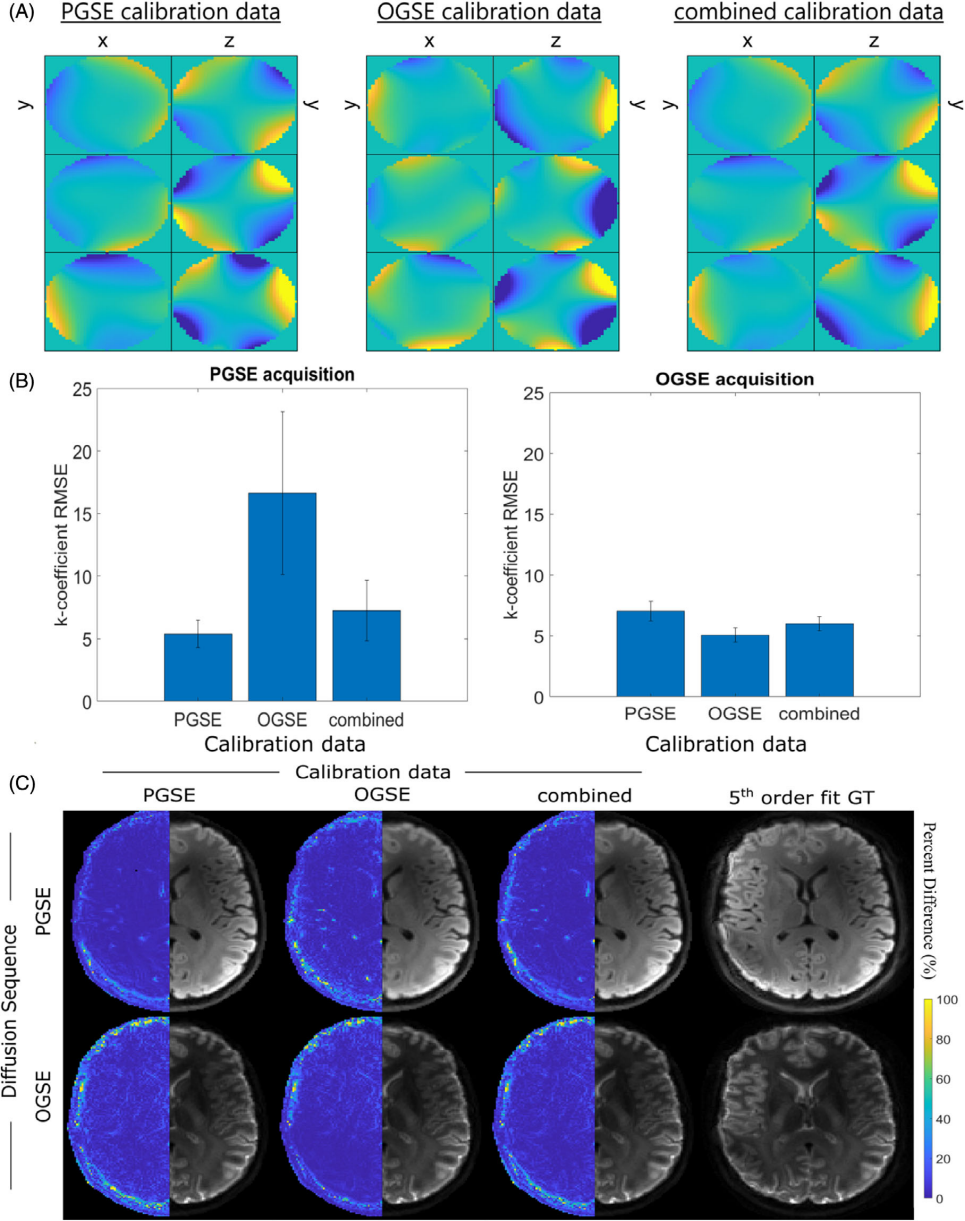
(A) Cross‐sectional spatial distribution (left‐to‐right: x‐y, y‐z planes) of compressed basis functions acquired from calibration data including pulsed gradient spin echo (PGSE), oscillating gradient spin echo (OGSE), and combined acquisitions. Rows represent the new basis functions related to the first three principal components. (B) Mean root‐mean‐square‐error (RMSE) analysis of up‐to‐second‐order k‐coefficients, calculated using the calibration data defined from PGSE, OGSE, and combined acquisitions. Comparison was performed for field dynamics measured for the PGSE acquisition (left) and OGSE acquisition (right). (C) Mean DWI reconstructions for the PGSE (top) and OGSE (bottom) acquisitions, when informed by field dynamics compressed based on the described calibration data: Left‐to‐right PGSE acquisition, OGSE acquisition, and combined diffusion data, plus respective fifth order fits calculated using 100 probes. Percent difference images were calculated relative to images informed by fifth order field dynamics and are shown in the left hemisphere of the images. Five singular values were kept for compressions, except for calibrations containing only OGSE data, where three singular values were preserved.

Differences in compressed basis function maps were observed between PGSE and OGSE calibration data (Figure 5A), yet there was strong similarity between the combined and PGSE cases. Accordingly, RMSE was highest for PGSE acquisitions that used OGSE calibration data, whereas the combined calibration data performed relatively well for both cases. (Figure 5B). These errors were observed as subtle blurring in the OGSE‐calibrated mean PGSE DWI (Video S2; Figure 5C).

**FIGURE 6:**
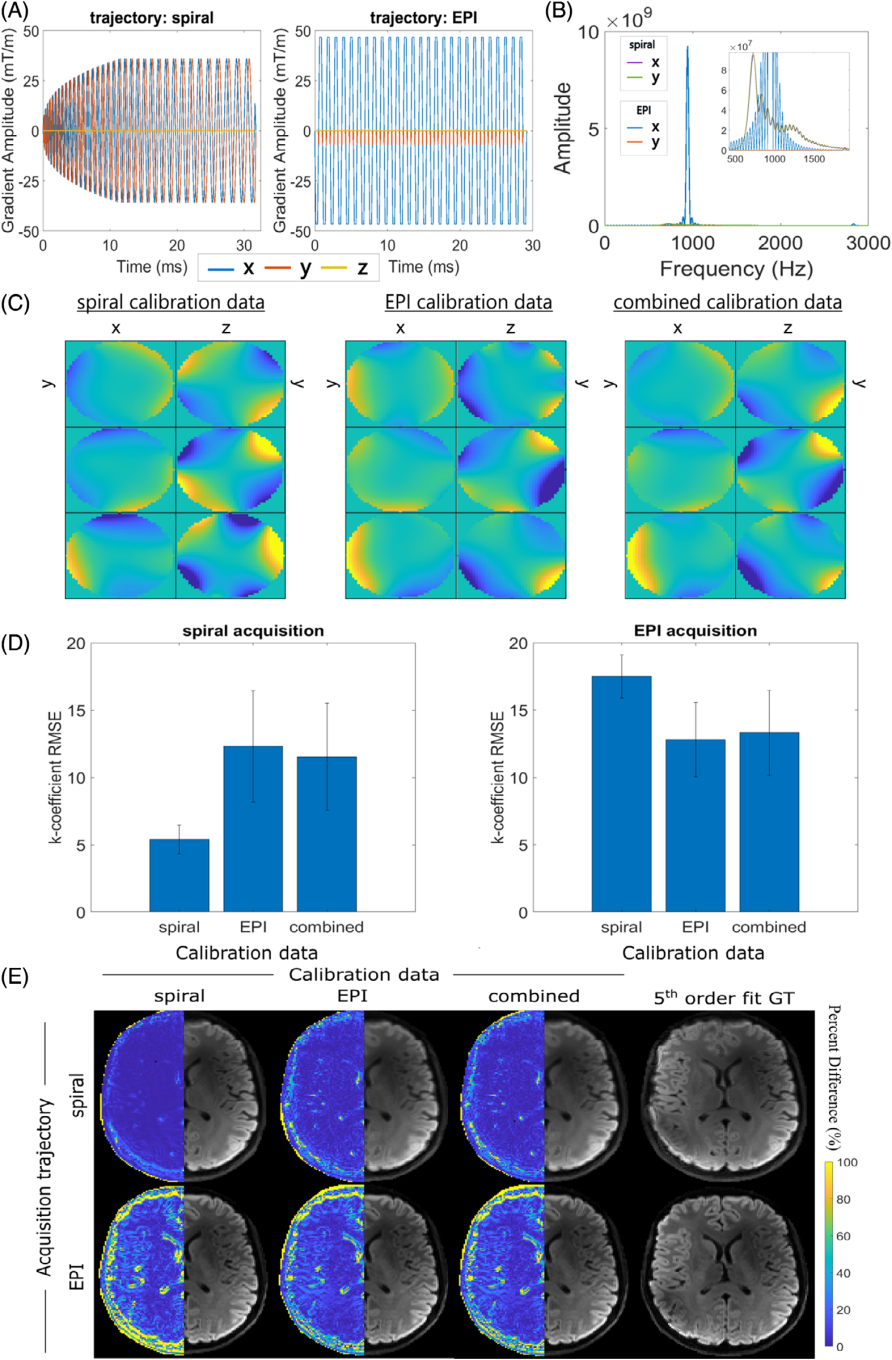
(A) Gradient readout profiles for the spiral and EPI acquisitions, and (B) respective power spectral density profiles. (C) Cross‐sectional spatial distribution (left‐to‐right: x‐y, y‐z planes) of compressed basis functions determined from calibration data using the spiral trajectory, EPI trajectory, and combined trajectories. Rows represent the new basis functions related to the first three principal components. (D) Mean root‐mean‐square‐error (RMSE) analysis of up‐to‐second‐order k‐coefficients, calculated using the calibration data from spiral, EPI, and combined acquisitions. Comparison was performed for field dynamics measured for the spiral acquisition (left) and EPI acquisition (right). (E) Mean DWI reconstructions for the spiral (top) and EPI (bottom) acquisitions, when informed by field dynamics compressed based on the described calibration data: left‐to‐right spiral acquisition, EPI acquisition, combined trajectories, plus respective fifth order fits calculated using 100 probes. Percent difference images were calculated relative to images informed by fifth order field dynamics and are shown in the left hemisphere of the images. Five singular values were kept for compressions, except for spiral reconstructions calibrated using EPI data, which preserved four singular values.

Gradient profiles for the spiral and EPI trajectories are illustrated in Figure 6A. Frequency content analysis showed relatively larger spectral energy along the x‐channel for the EPI trajectory, overlapping significantly with both channels of the spiral trajectory (Figure 6B). Accordingly, more substantial higher order phase evolution was observed for the EPI acquisition (Figure S3A). Although the improvements in k‐coefficient error were significant using the compressed method, the reduction in error was far less than for the spiral acquisition (Figure S3B). Qualitative (Figure S3C) and quantitative (Figure S3D) image analysis did not present improvements in image reconstruction performance using the compressed method, given the manifestation of the lower order errors as pronounced image shifts. However, when using 32 probes (subset of 100‐probe array) instead of the conventional 16 for EPI, a higher number of singular values (nine vs. five) achieved minimal error and performance is considerably improved (Figure S3). When investigating combination of spiral and EPI calibration scans with the standard 16‐probe array, there was strong similarity between the combined case and the EPI trajectory (Figure 6C). RMSE values were the lowest when using calibration data matching the image reconstruction, but the error for the spiral acquisition was considerably lower than for EPI (Figure 6D). The combined calibration performed poorly for spiral compared to EPI. Small but noticeable qualitative differences in mean DWI were observed when calibrating with the other trajectory or for calibrating with the combined data for spiral (Figure 6E).

**FIGURE 7:**
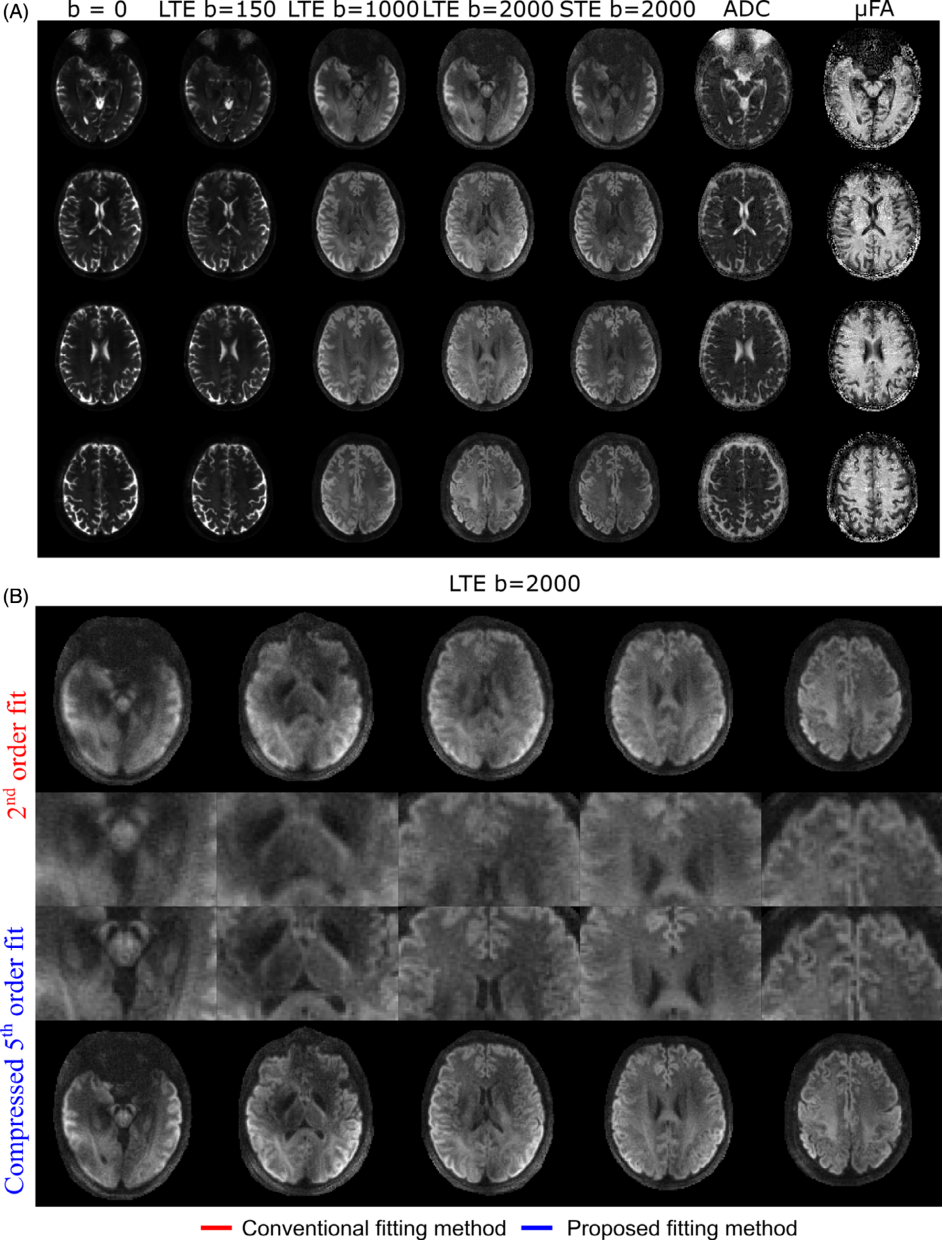
(A) Mean sample image slices reconstructed for the linear and spherical tensor‐encoded acquisitions incorporated in the b‐tensor encoding scan, as well as computed ADC and μFA maps. Reconstructions were informed with compressed fifth order field dynamics, using the compression matrix determined from “scan 1” calibration data. (B) Comparison of mean DWI from the linear tensor‐encoded (LTE) scan (*b* = 2000 s/mm^2^) for reconstructions informed with conventional second order fits (top) and compressed fifth order fits (bottom), with zoom‐ins highlighting the blurring reduction observed when implementing compressed fifth order fits.

The b‐tensor encoding scan reconstructed using the calibration matrix determined from scan 1 resulted in DWIs and ADC/μFA maps with high quality throughout the brain (Figure 7A). The high‐quality STE images suggest good generalizability from LTE encodings used in the calibration. Mean DWI acquired from the LTE encoding scheme exhibited large improvements in image quality in comparison to images that were reconstructed with the conventional second order fit (Figure 7B).

**FIGURE 8:**
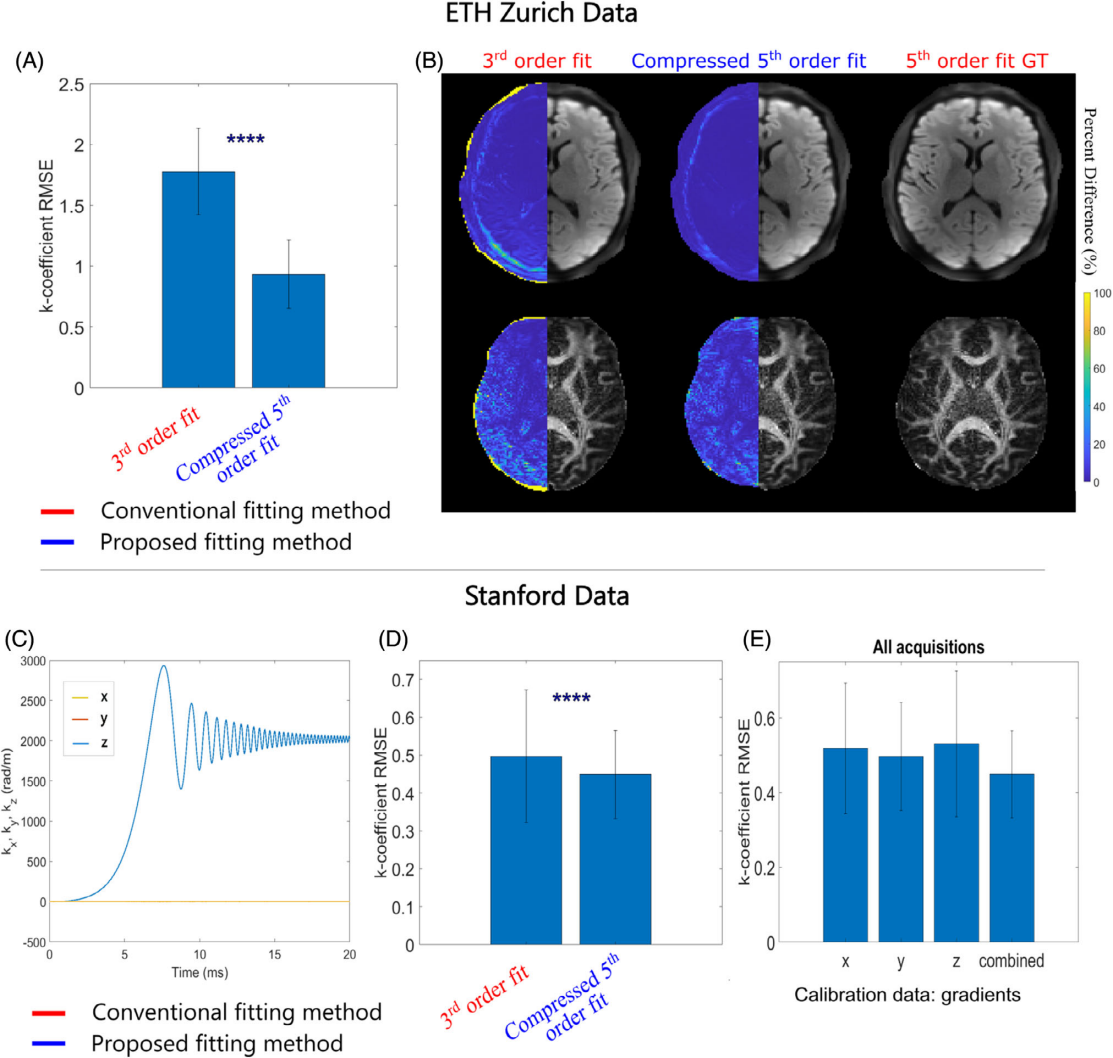
Algorithm performance on data from two additional scanners: Philips 3 T housing a high‐performance gradient coil (top), and GE 3 T Ultra‐High‐Performance scanner (bottom). (A) Mean root‐mean‐square‐error (RMSE) comparison of k‐coefficients up‐to‐second order when performing a conventional third order fit, and a compressed fifth order fit, relative to fifth order field dynamics computed using 64 probes. (B) Reconstructed mean DWI and calculated fractional anisotropy (FA) maps when informed by field dynamics determined by the same fitting schemes. Percent difference images were calculated relative to images informed by fifth order field dynamics and are shown in the left hemisphere of the images. Seven singular values were kept for compression. (C) First‐order field‐monitored trajectories from the chirped acquisition, for a sweep along the z‐axis. (D) Mean RMSE comparison of k‐coefficients up‐to‐second order when performing a conventional third order fit, and a compressed fifth order fit, relative to fifth order field dynamics computed using 48 probes. (E) Mean RMSE of k‐coefficients as a function of the gradient frequency sweep acquisitions (x, y, z) included in the calibration data. Data up to 20 ms was included in the error analysis. Four singular values were kept for compression.

Analysis of k‐coefficient profiles calculated from the ETH Zurich scan showed good agreement between compressed fifth order terms and ground truth fifth order terms (Figure S4A), and a significant reduction in overall error was observed when using the proposed fitting method as opposed to conventional third order fit (Figure 8A). Accordingly, mean DWI and FA maps informed by compressed fifth‐order k‐coefficients showed better agreement with ground truth reconstructions than conventional third order fits (Figure 8B). For the frequency‐sweep acquisition from Stanford (Figure 8C), compressed fifth order k‐coefficient data also showed better agreement with the ground truth profiles when compared to conventional third order (Figure S4B), which resulted in an overall reduction in mean RMSE error (Figure 8D). That said, the reduction in RMSE is modest, likely because there are few higher order terms for the large body gradient compared to the other specialized gradient systems investigated in this work. A combination of all the frequency‐sweeps from all three gradient channels provided the best compression performance, as opposed to calibrations of individual channels (Figure 8E).

**FIGURE 9:**
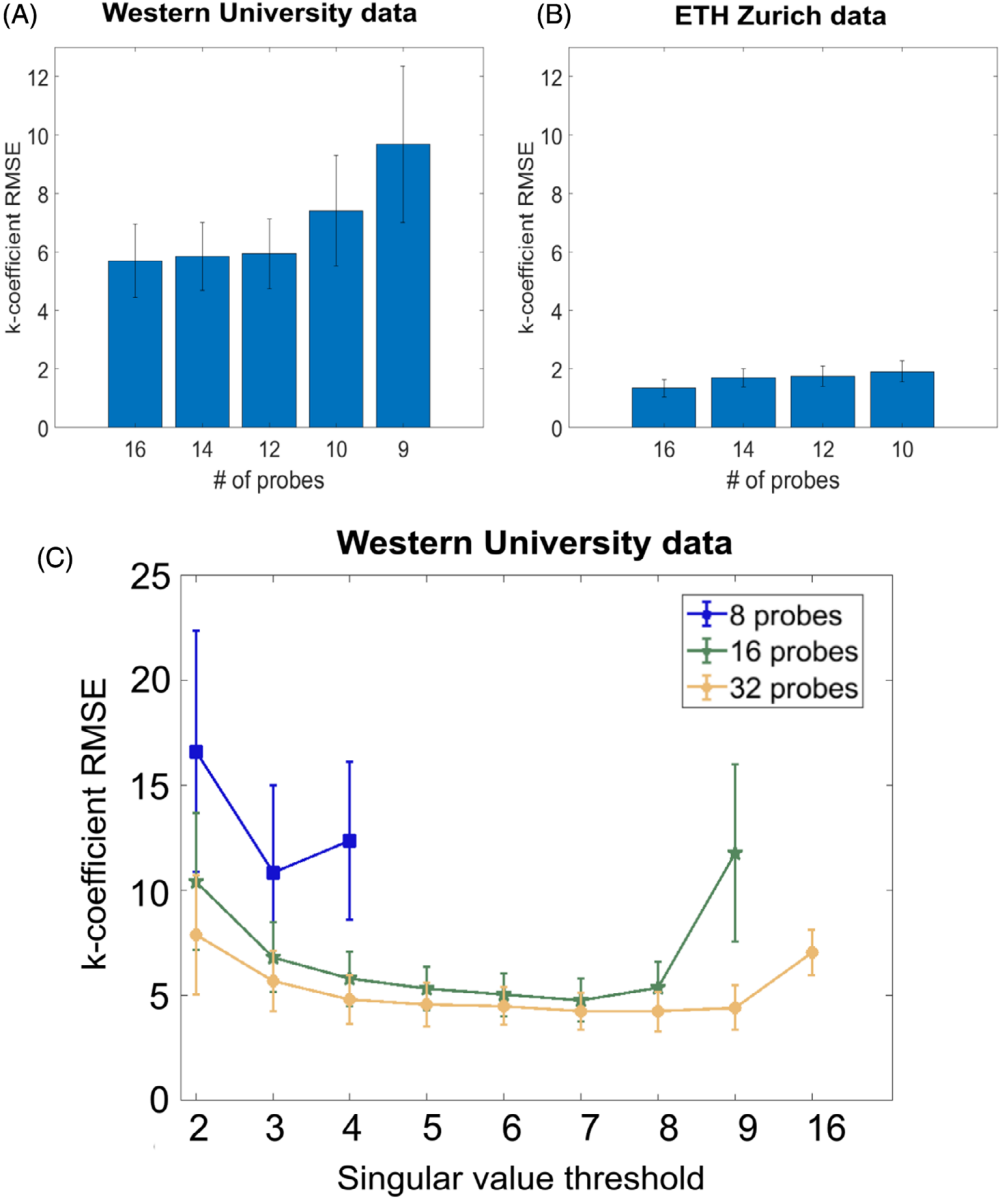
Compression evaluation based on probe input for Western University and ETH Zurich data. (A) k‐Coefficient mean root‐mean‐square‐error (RMSE) analysis for a range of 9–16 probes used for compressed fitting for Western University data. Five singular values kept resulting in a total of nine basis functions, hence, the lowest probe amount equaling nine. (B) k‐Coefficient mean RMSE analysis for a range of 10–16 probes used for compressed fitting for ETH Zurich data. Six singular values kept resulting in a total of 10 basis functions, hence, the lowest probe amount equaling 10. (C) k‐Coefficient RMSE evaluation as a function of singular value quantity, for probe fitting amounts of eight, 16, and 32 probes. Further points are not plotted for eight probes and 16 probes because of the total number of basis functions exceeding the number of probes. Probe subsets were determined by maximizing electrostatic repulsion for each subset.

Compression performance based on the probe quantity showed incremental increases in RMSE as the probe quantity was reduced for compressed fitting (Figure 9A,B), with larger increases occurring specifically below 12 probes for the Western data (Figure 9A). When performing singular value optimization tests using RMSE for initial inputs of eight, 16, and 32 probes, the RMSE was more sensitive to the addition of more singular values when fewer probes were used. When 32 probes were used, more singular values could be retained before seeing a noticeable increase in error (Figure 9C).

## 4 DISCUSSION

In this work, we introduced a field monitoring procedure aimed to accurately fit higher order phase accrual using fewer field probes than are typically required. This was motivated by the need to improve field dynamic characterizations on systems that exhibit substantial higher spatial modes, such as high‐performance gradients and integrated field probe setups. The technique requires a calibration scan that involves rotations and/or translations of the field probe array around the imaging volume, followed by the creation of a compression matrix.

### 4.1 Truncation trade‐offs

As more singular values are included in a low rank approximation of a matrix, the error of that approximation will decrease. However, here, we find that the RMSE of the k‐coefficients determined from that approximated matrix increases beyond some optimal number of retained singular values (Figure 2A). This result, which seems unexpected on the surface, likely occurs because there is a trade‐off between preserving enough significant singular values to maintain accuracy of the basis function representation while avoiding including too many that the number of unknown k‐coefficients approaches the number of probes. With too few singular values, k‐coefficient RMSE and image degradation increases because of the removal of important basis functions for the acquisition. However, when the total number of basis functions is close to the number of probes, $\hat{P}$ becomes more poorly conditioned leading to increased error when solving Eq. 9. This trade‐off is even more evident in Figure 9C and Figure S3D, where it is observed that for more probes the optimal number of retained basis functions increases and k‐coefficient errors decrease.

### 4.2 Global generalizability

Assuming that the eddy current response is linear time‐invariant, it is expected that a single comprehensive calibration scan that covers all bandwidths and amplitudes of expected gradients could be used to determine a general set of compressed basis functions that would be applicable to all scans. The Stanford results suggest this could be applicable, considering that even for the collection of very large bandwidth chirp scans, k‐coefficient fits with compressed basis functions and 16 probes resulted in decreased RMSE for k‐coefficient estimations. However, the ability to use a globally general calibration depends on the number of significant compressed basis functions relative to the number of available probes, as discussed in the preceding paragraph, with the required surplus also depending on the arrangement of the probe array. The Western results suggest that more than the standard 16 probes are needed for acquisitions that produce significant higher order behavior such as the EPI sequence used (Figure S3) or for a calibration that combines multiple differentiable scans. To explore this further, we combined all calibration data (scans 1–4) and found that a 32‐probe subset was needed for this global calibration data set to match the accuracy of an axial‐only spiral calibration scan (Figure S5B), as more singular values needed to be retained given the presence of more significant singular values (Figure S5A). The presence of many significant spatial modes that require more than the standard 16 probes typically included with a field probe system suggests global calibration is not possible for the Western setup. The higher contributions from high‐order modes likely stem from all probes lying far from isocenter (>10 cm), well outside the DSV, where higher‐order terms become more significant. The Stanford data did not have this issue, because it is a full body system with the probes being in ideal positions less than 10 cm from isocenter. The ETH Zurich results also show excellent compression performance with approximately 5 times lower k‐coefficient RMSE than the Western results, which was likely due, in part, by the optimal probe placement near isocenter, allowing a higher singular value count of seven to be effectively compressed with the standard 16 probe array. Accordingly, this work illustrates the potential drawbacks in integrating field probes into RF hardware, whereas it enables simultaneous field monitoring, and it opens the door to errors from higher spatial orders during k‐coefficient fitting.

### 4.3 Calibration considerations

When important basis functions are more numerous than the number of available probes, basis function compression can provide insight into spatial modes that have low weights and can be ignored during k‐coefficient fitting, which reduces the minimum number of probes required. However, this requires that a calibration scan comparable to the scan of interest should be used, so that spatial modes with low weights in the scan of interest also have low weights in the calibration data. Integrating the results from Figures 3, 4, 5, it is evident that a single‐shot spiral calibration scan that (1) is high resolution, (2) uses PGSE diffusion gradients, and (3) uses at least six uniformly distributed diffusion gradient directions will be generalizable to nearly any single‐shot spiral diffusion MRI (dMRI) scan on the Western system and still have few enough spatial modes that 16 probes are sufficient. This assertion is supported by the good performance shown in Figure 7, which used a different resolution, diffusion gradient waveform shape (i.e., b‐tensor encoding), diffusion weighting, and set of diffusion directions compared to the calibration scan (only scan 1 was used to generate compressed basis functions for the acquisition shown in Figure 7). Accordingly, basis function compression can be relatively broadly applied to spiral dMRI on our system with a single set of calibrated basis functions, even though a global calibration is infeasible. Although the Western system is relatively unique, high‐performance head‐only gradient systems^22^, ^23^, ^42^ are being heavily invested into for neuroscience applications, and basis function compression may also be needed for these types of systems. This is especially true if simultaneous field monitoring is sought, because it would require moving the probes far from isocenter. For more standard configurations where there are fewer important modes than probes, there is likely a benefit to using global compressed basis functions (e.g., determined from a comprehensive collection of chirped and diffusion gradient waveforms) over spherical harmonics, as evidenced by the Stanford results, because this guarantees that the spatial modes that are most significant for any given system are being prioritized.

It may be possible to apply weights to compressed basis functions according to their expected significance during k‐coefficient fitting to further improve accuracy (e.g., based on singular values obtained during compression). It may also be possible to perform a comprehensive global calibration scan and then retrospectively determine which basis functions should be used for a particular scan of interest, which would mitigate the somewhat prohibitive requirement to have calibration scans tailored to “classes” of scans (e.g., single shot spiral or EPI). However, investigation of such extensions to this work is beyond the scope of this initial investigation that aims to introduce the theory behind basis function compression and demonstrate its potential utility. Finally, although manual determination of the optimal number of singular values was determined by comparison to a ground truth with many probes, the development of less user‐intensive means of determining the threshold could be an area of future work.

### 4.4 Other observations

The improved higher order mode characterization and generalizability of PGSE compared to OGSE is likely because of PGSE exhibiting a broader spectral response, whereas OGSE excites a finite frequency range. Additionally, OGSE has been shown to exhibit reduced higher order behavior, likely stemming from the partial canceling of the higher order modes from the alternating positive and negative gradient ramps.^10^, ^43^ A relatively poor performance of basis function compression for EPI was observed compared to spiral, which stemmed from the excitation of many spatial modes that require more than 16 probes to characterize (Figure S3). This may have stemmed, in part, from the higher gradient amplitude of the EPI waveform that was chosen to match the resolution of the spiral acquisition, whereas maintaining a short enough readout time to avoid significant probe signal decay. Nevertheless, non‐Cartesian sequences like spiral have the strongest need for field monitoring because of more severe artifacts that result from eddy current fields,^44^, ^45^ and for this application the results in this work are encouraging.

### 4.5 Fewer probes

The potential to reduce the probe amount required for high order fitting, below the 16 provided commercially, may be advantageous in certain scenarios. Namely, when integrating field probes into RF coils, challenges in probe placement can arise especially when handling complex coils that house many elements. Fewer probes may simplify the incorporation of all probes and required elements. Additionally, instances may arise where only select probes are subject to rapid field variations. This technique enables removal of unfavorable probes that may hinder phase fitting, whereas still permitting higher order characterizations. However, as previously discussed, a further reduction in probes may also limit the accurate compression of calibration sets that exhibit more pronounced or generalized higher order modes, which should be taken into consideration.

### 4.6 Comparison to other approaches

The proposed technique was also compared to a previous approach presented by the authors, which in addition to weighted least squares fitting discussed earlier fit the spatial orders one at a time to reduce error in lower order terms.^24^ Although application of this previous technique resulted in reduced RMSE error and DWI blurring, further significant improvements were observed using basis function compression (Figure S6).

### 4.7 Limitations

The calibration scheme did not consider spatial modes that might arise from physiological processes during concurrent monitoring, such as field changes from breathing. However, the differences are expected to be small because of the use of rapid single‐shot trajectories. Additionally, for the Western data, a chirped calibration scan was not considered. However, given that we already show challenges for generalizability here because of limitations in characterizing higher order information, it is unlikely that such a calibration scan with a broad range of frequencies and gradient directions would perform well for our system where compression works best with significantly fewer total basis functions than the standard probe amount. It is also worth noting that acquiring field monitoring measurements in various probe positions may be challenging, particularly when using integrated field probe coils. For our configuration, modifications to cable connections and removal of the patient cradle were necessary to allow for relatively unrestricted movement of the head coil within the imaging volume. Lastly, it is likely that changing the RF coil position can alter the eddy current patterns because of reconfiguration of RF shielding components, particularly if asymmetric shielding is used. Although not a large concern for the head‐only scanner because of the absence of shielding in the head coil, this may be worth investigating on other systems by assessing the degree of field dynamic changes with reconfiguration of the RF coil.

## 5 CONCLUSION

The presented field monitoring strategy enables the incorporation of additional higher order information than is permitted with a conventional collection of probes. This results in improvements in accuracy of field dynamics and diffusion data, specifically when field probes experience rapid spatial field variations. For the setup at Western with field probes integrated into the RF coil far from isocenter, standard spherical harmonic representation of k‐coefficients resulted in unusable single‐shot spiral images with severe artifacts. In contrast, compressed basis functions enabled high‐quality single‐shot spiral dMRI, with a compression calibration that can be broadly applied to nearly any spiral dMRI scan. With the rise of specialized gradient systems having high gradient strengths and slew rates, field probes become more attractive to account for higher order perturbations. Using this technique to accurately measure these higher spatial orders may improve the ability to tap into the advanced capabilities of these systems, while maintaining high‐quality image production.

## CONFLICT OF INTEREST STATEMENT

K.P.P. holds a research agreement with and receives research support from Philips and is a shareholder of Gyrotools.

## Supporting information


**Data S1.** Scan details for additional sites.
**Figure S1.** Reconstructed mean DWI informed by compressed k‐coefficients calculated from “Scan 1” calibration data, using the described number of singular values, for the complete range of 2–9 singular values investigated. Comparable image quality was observed in the range of 4–7 singular values, whereas use of 2–3 and 8–9 singular values introduced significantly more blurring.
**Figure S2.** Sample 0th–5th order k‐coefficient time‐courses of the 1.3‐mm single‐shot spiral acquisition (Scan 1), for different fitting methods: fifth order fit using 100 field probes (black), compressed fifth order fit (red), conventional first order fit (blue), and conventional second order fit (magenta). Overall, better agreement in first order terms was observed between the ground truth fifth order fit and compressed fifth order fit methods. Good agreement in these methods was also seen for the higher order terms.
**Figure S3.** EPI compression performance. (A) Comparison of the higher order k‐coefficients for the 1.3‐mm spiral acquisition (Scan 1, black) and 1.3‐mm EPI acquisition (Scan 4, red), for which the EPI acquisition exhibited stronger higher order behavior. (B) illustrates the error for first‐order k‐coefficients relative to the ground truth (fifth order fit using 100 probes) for conventional first and second order fits and compressed fifth order fit using 5 singular values (SV) and the nominal 16 probes, as well as 9 singular values with a 32‐probe subset. (C) Respective reconstructed mean DWI and calculated fractional anisotropy (FA) maps when incorporating the same fitting schemes. Percent difference images were calculated relative to images informed by fifth order field dynamics and are shown in the left hemisphere of the images. (D) Respective normalized‐root‐mean‐squared‐error (NRMSE) and structural similarity index (SSIM) comparisons of reconstructed image volumes and fractional anisotropy (FA) maps relative to the ground truth images. For the standard setup, compression performance underperforms for this particular EPI acquisition due to the presence of substantial higher order behavior that isn't accurately characterized by the inclusion of only the first 5 singular values. As a result, the fitting errors significantly manifest in the lower order terms. While the compressed k‐coefficient error is still lower than the conventional fits, the resulting bulk image shifts (illustrated in c) translate to the larger image errors reported by the NRMSE and SSIM. Doubling the initial probe amount to 32 enabled the inclusion of other substantial singular values, which resulted in significantly improved compression performance, as evidenced by the lower k‐coefficient error, and improved qualitative and quantitative image reconstructions and FA maps (SSIM close to 1).
**Figure S4.** Sample 0th–5th order k‐coefficient time‐courses of the single‐shot spiral acquisition from (A) ETH Zurich and (B) the frequency sweep (Stanford), for different calculation methods: fifth order fit using 64 or 48 field probes (black), compressed fifth order fit (red), and conventional first order fit (blue). In both cases, better overall agreement was observed between the ground truth and compressed fifth order fit techniques, especially for second and third order terms.
**Figure S5.** (A) Singular value distributions for the following calibration datasets: 1.3‐mm spiral acquisition “Scan 1” (blue) and for the combined calibration acquisitions “Scans 1–4” (red), with the combined calibration data exhibiting more prominent singular value weights. (B) Illustrates mean k‐coefficient root‐mean‐square‐error (RMSE) (in blue) for the spiral acquisition (using Scans 1–4 combined calibration data) as a function of the following probe subset quantities provided during compressed fitting: 16, 24, 32, 48, 64. Probe subsets were determined by maximizing electrostatic repulsion from the 100‐probe array. The red graph represents the respective number of singular values retained for compression following minimization of the RMSE: 7, 9, 9, 9, and 15. With enough probes, the number of singular values that minimizesthe RMSE increases, thereby also substantially reducing the RMSE. This suggests that the additional singular values possess valuable higher order modes. When reducing the number of preserved singular values, due to the constraints imposed by fewer probes, the error substantially increases, with the largest error observed for the 16‐probe arrangement. The consistent minimization using 9 singular values when decreasing from 48 to 24 probes may be due to the importance of the 9th singular value, as can be seen in the singular value plot. While preserving 9 singular values amounts to 13 total basis functions, which is still below the probe amount for the 16‐probe case, fewer singular values (7) minimized the error likely because the poorer conditioning of the probing matrix when including more basis functions counteracts the benefits of including these significant singular values. Accordingly, compression performance proves to be limited for the Western setup when including differentiable calibration datasets.
**Figure S6.** Comparison of different proposed fitting techniques. (A) Quantitative k‐coefficient root‐mean‐square‐error (RMSE) analysis up‐to‐second order and (B) resulting qualitative mean DWI comparison informed by field dynamics computed conventionally with no form of fitting correction (left), using a previous fitting approach proposed by the authors (middle), and using the compressed basis function fitting approach (right). Improvements in both k‐coefficient similarity and blurring reduction were observed with each successive iteration of fitting algorithm implemented.

**Video S1.** Video comparison of mean DWI and fractional anisotropy (FA) maps informed by conventional third order fits using 16 probes, third order fit using 100 probes, and fifth order fit using 100 probes. Significant improvements in image quality were apparent when moving to third order fit with a probe surplus. Small reductions in blurring in the DWI and improvement in the FA map were also observed when implementing a fifth order fit.

**Video S2.** Mean DWI from a spiral pulsed gradient spin echo (PGSE) acquisition informed with calibration data from the PGSE acquisition, oscillating gradient spin echo (OGSE) acquisition, and when combining the two diffusion acquisitions. Blurring becomes more apparent when the compression matrix was determined using data from the OGSE acquisition.

## Data Availability

The basis function compression technique and the source code used for the expanded encoding model reconstructions are publicly available in the MatMRI toolbox: https://zenodo.org/records/5708265 and https://gitlab.com/cfmm/matlab/matmri. A demo calculating k‐coefficients from raw probe data measured from a spiral acquisition on the head‐only 7 T with and without the proposed fitting scheme is included.
