# Supplementary material for: Basis function compression for field probe monitoring
Source: Magn Reson Med. 2025 Feb 18;93(6):2414–33. doi: 10.1002/mrm.30471 (PMC11971494; doi:10.1002/mrm.30471)
Supplement: Supplementary file 1 — Data S1. Scan details for additional sites. Figure S1. Reconstructed mean DWI informed by compressed k‐coefficients calculated from “Scan 1” calibration data, using the described number of singular values, for the complete range of 2–9 singular values investigated. Comparable image quality was observed in the range of 4–7 singular values, whereas use of 2–3 and 8–9 singular values introduced significantly more blurring. Figure S2. Sample 0th–5th order k‐coefficient time‐courses of the 1.3‐mm single‐shot spiral acquisition (Scan 1), for different fitting methods: fifth order fit using 100 field probes (black), compressed fifth order fit (red), conventional first order fit (blue), and conventional second order fit (magenta). Overall, better agreement in first order terms was observed between the ground truth fifth order fit and compressed fifth order fit methods. Good agreement in these methods was also seen for the higher order terms. Figure S3. EPI compression performance. (A) Comparison of the higher order k‐coefficients for the 1.3‐mm spiral acquisition (Scan 1, black) and 1.3‐mm EPI acquisition (Scan 4, red), for which the EPI acquisition exhibited stronger higher order behavior. (B) illustrates the error for first‐order k‐coefficients relative to the ground truth (fifth order fit using 100 probes) for conventional first and second order fits and compressed fifth order fit using 5 singular values (SV) and the nominal 16 probes, as well as 9 singular values with a 32‐probe subset. (C) Respective reconstructed mean DWI and calculated fractional anisotropy (FA) maps when incorporating the same fitting schemes. Percent difference images were calculated relative to images informed by fifth order field dynamics and are shown in the left hemisphere of the images. (D) Respective normalized‐root‐mean‐squared‐error (NRMSE) and structural similarity index (SSIM) comparisons of reconstructed image volumes and fractional anisotropy (FA) maps relative to the ground truth image [file MRM-93-2414-s002.docx]

**Supporting Information**

**Supporting Information S1** Scan details for additional sites

ETH Zurich Data

A healthy volunteer was scanned on a 3T Philips Achieva scanner housing a high-performance head-only gradient insert (200 mT/m max gradient strength and 600 T/m/s max slew rate, [22, 22, 20 cm] imaging volume in x, y, z directions respectively)^1^ after approval from the institutional review board. A single-shot spiral diffusion-weighted acquisition was performed with the following imaging parameters: FOV: 220 x 220 mm^2^, 1.5 mm in-plane resolution, 3 mm slice thickness, number of slices: 20, TE/TR: 21/6,000 ms, rate 2 undersampling, bandwidth = 222 kHz, b = 0 s/mm^2^ acquisitions: 2, diffusion directions: 6, b-value: 1000 s/mm^2^ using a PGSE scheme, axial orientation. Data for coil sensitivities and B_0_ maps was acquired using a multi-echo gradient echo scan. Using a dynamic field camera (Skope, Zurich, Switzerland), field monitoring of an identical imaging acquisition was performed in 4 different field camera positions, with each position involving a rotation of the camera about the z-axis, and one position being slightly translated along the z-direction. From this, a probe array consisting of 64 probes was compiled. A ground truth fifth-order fit was performed for the acquisition, followed by the determination of a truncated compression matrix. Using this matrix, a compressed fifth order fit and a conventional third order fit were performed using only the 16 probes from the first orientation, mean probe distance from isocenter = 8.4 cm (range: 7.7–8.9 cm). Images were reconstructed using an in-house model-based reconstruction algorithm at ETH Zurich.^2^

Stanford Data

A chirped scan was performed on the GE 3T Ultra-High-Performance scanner (100 mT/m max gradient strength, 200 T/m/s max slew rate, 50-cm imaging volume) at Stanford University’s Center for Cognitive and Neurobiological Imaging. The scan involved repeated acquisitions of frequency modulations from 0 to 26 kHz individually along the x, y, and z gradients. A total of 48 sweeps were repeated along each gradient axis. Using a dynamic field camera (Skope, Zurich, Switzerland), field monitoring of the acquisition was performed in an empty scanner repeatedly for three different probe orientations, where the field probes were rotated about the z-axis in each case. Using this data, a probe array consisting of 48 probes was compiled, and a ground truth fifth order fit of the acquisition was performed. A compression matrix was determined using the calibration data. The singular value threshold was determined based on minimized RMSE of the k-coefficients relative to the ground truth. While frequency sweeps were 75 ms in duration, only data points up to 20 ms were included in the calibration as this comprised much of the high-amplitude gradient oscillations, and limited the amount of noisy data points that could propagate into the principal component analysis. A compressed fifth order fit and a conventional third order fit were performed using the conventional 16-probe arrangement, mean probe distance from isocenter = 8.5 cm (range: 7.9–8.8 cm).

**References**

1. Weiger M, Overweg J, Rösler MB, et al. A high-performance gradient insert for rapid and short-T2 imaging at full duty cycle. *Magn Reson Med*. 2018;79(6):3256-3266.
2. Wilm BJ, Barmet C, Pavan M, Pruessmann KP. Higher order reconstruction for MRI in the presence of spatiotemporal field perturbations. *Magn Reson Med*. 2011;65(6):1690-1701.


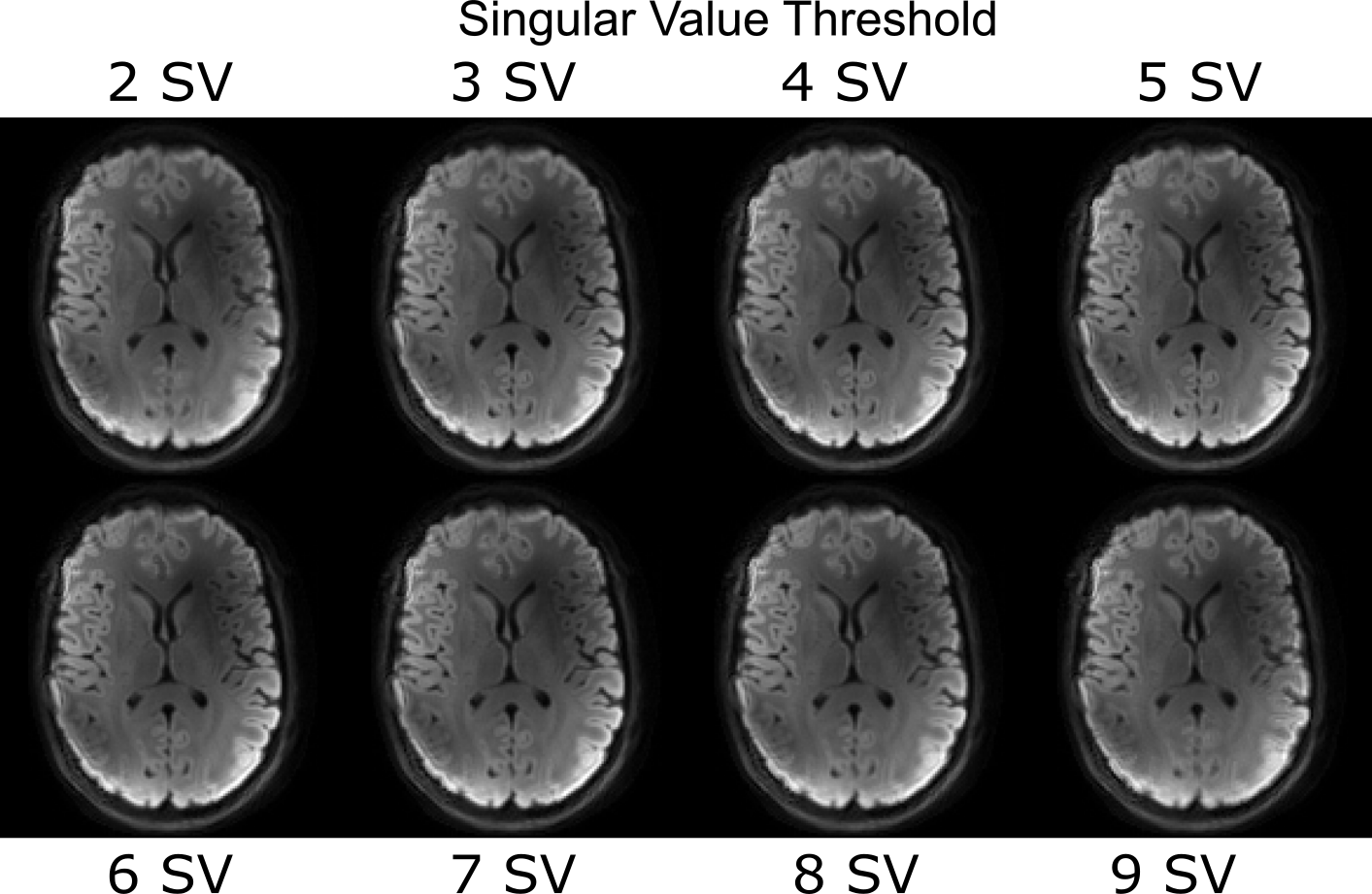


**Figure S1** Reconstructed mean DWI informed by compressed k-coefficients calculated from “Scan 1” calibration data, using the described number of singular values, for the complete range of 2-9 singular values investigated. Comparable image quality was observed in the range of 4-7 singular values, whereas use of 2-3 and 8-9 singular values introduced significantly more blurring.


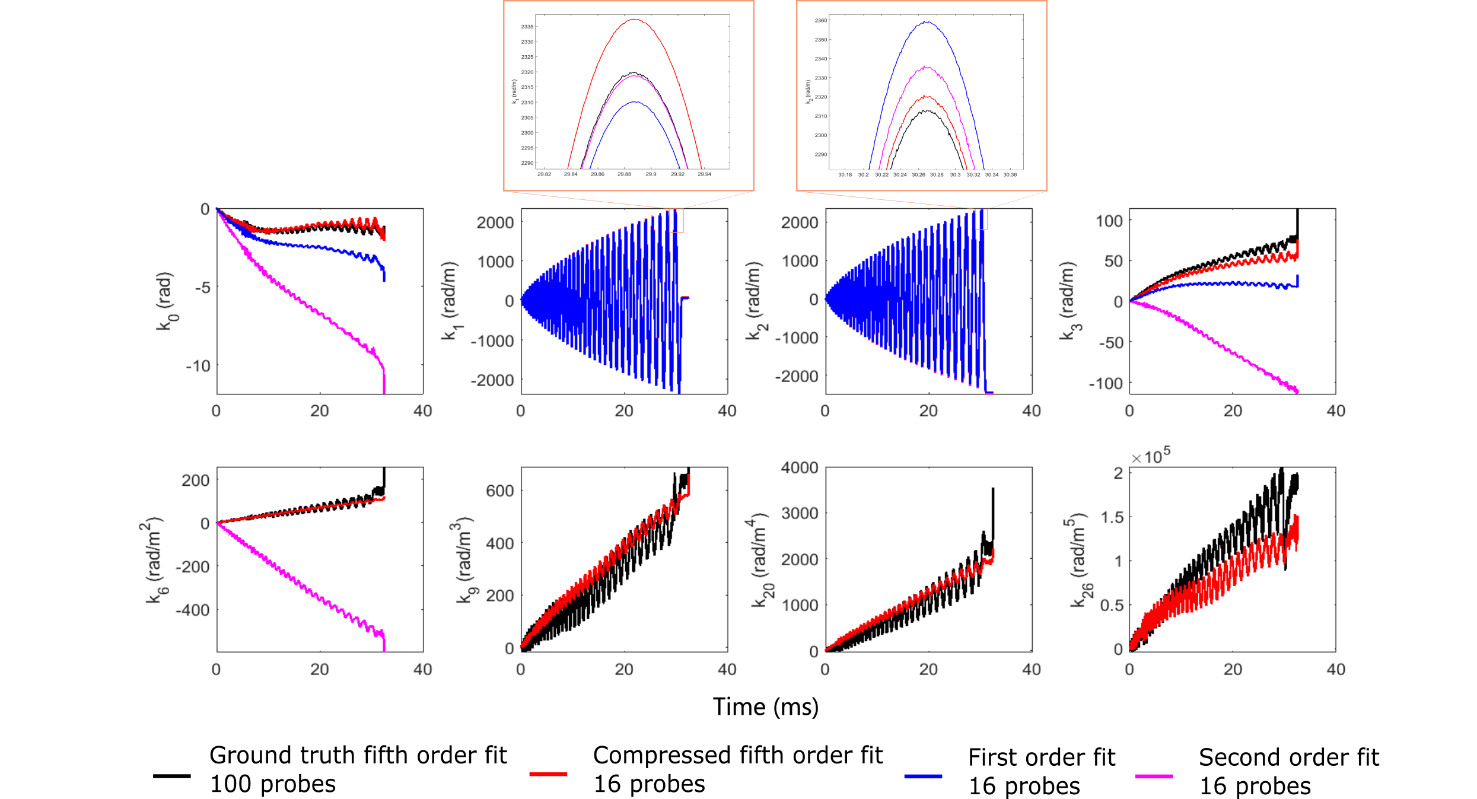


**Figure S2** Sample 0^th^-5^th^ order k-coefficient time-courses of the 1.3-mm single-shot spiral acquisition (Scan 1), for different fitting methods: fifth order fit using 100 field probes (black), compressed fifth order fit (red), conventional first order fit (blue), and conventional second order fit (magenta). Overall, better agreement in first order terms was observed between the ground truth fifth order fit and compressed fifth order fit methods. Good agreement in these methods was also seen for the higher order terms.


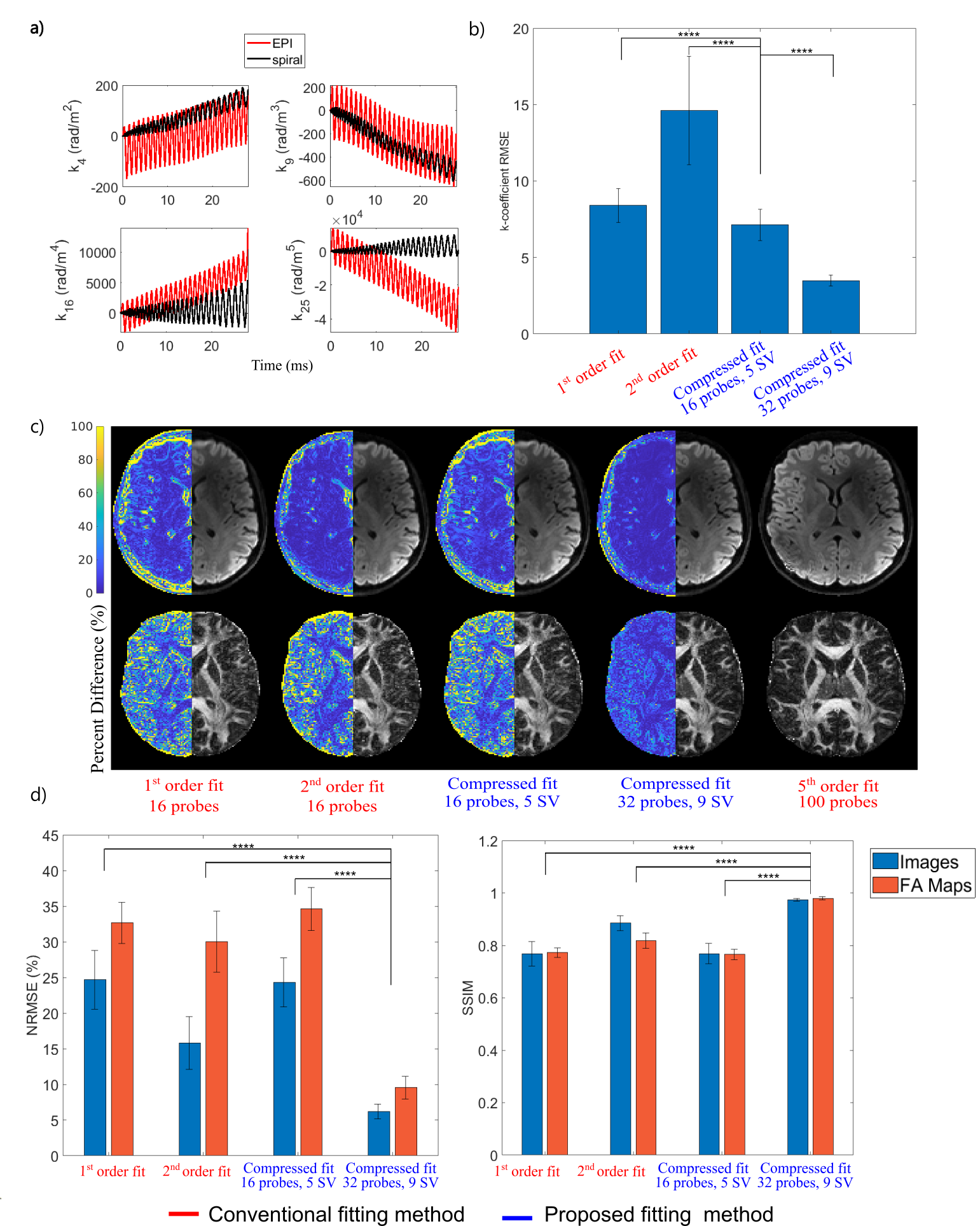


**Figure S3** EPI compression performance. (a) Comparison of the higher order k-coefficients for the 1.3-mm spiral acquisition (Scan 1, black) and 1.3-mm EPI acquisition (Scan 4, red), for which the EPI acquisition exhibited stronger higher order behaviour. b) illustrates the error for first-order k-coefficients relative to the ground truth (fifth order fit using 100 probes) for conventional first and second order fits and compressed fifth order fit using 5 singular values (SV) and the nominal 16 probes, as well as 9 singular values with a 32-probe subset. c) Respective reconstructed mean DWI and calculated FA maps when incorporating the same fitting schemes. Percent difference images were calculated relative to images informed by fifth order field dynamics and are shown in the left hemisphere of the images. d) Respective normalized-root-mean-squared-error (NRMSE) and structural similarity index (SSIM) comparisons of reconstructed image volumes and fractional anisotropy (FA) maps relative to the ground truth images. For the standard setup, compression performance underperforms for this particular EPI acquisition due to the presence of substantial higher order behaviour that isn’t accurately characterized by the inclusion of only the first 5 singular values. As a result, the fitting errors significantly manifest in the lower order terms. While the compressed k-coefficient error is still lower than the conventional fits, the resulting bulk image shifts (illustrated in c) translate to the larger image errors reported by the NRMSE and SSIM. Doubling the initial probe amount to 32 enabled the inclusion of other substantial singular values, which resulted in significantly improved compression performance, as evidenced by the lower k-coefficient error, and improved qualitative and quantitative image reconstructions and FA maps (SSIM close to 1).

**
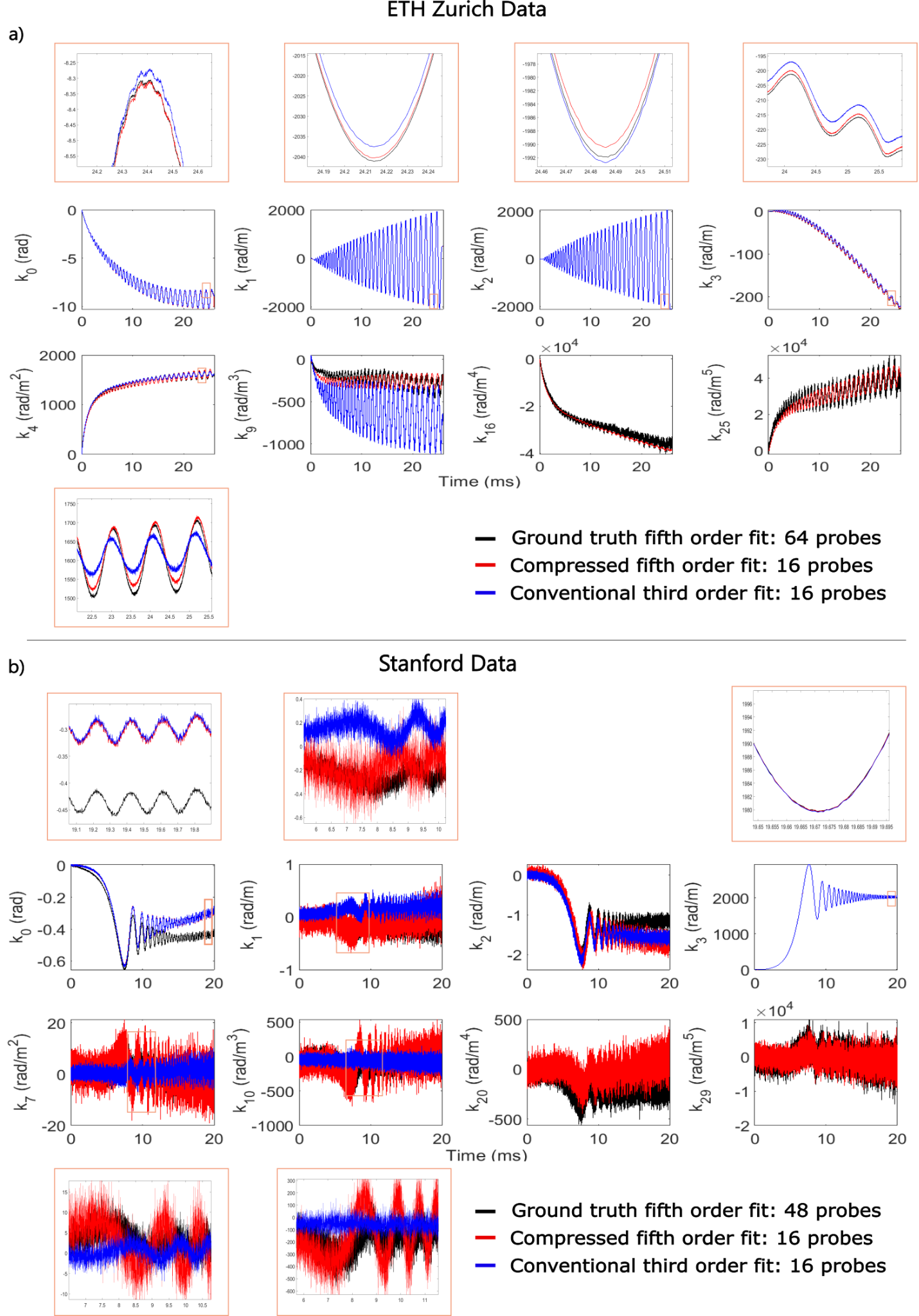
**

**Figure S4** Sample 0^th^-5^th^ order k-coefficient time-courses of the single-shot spiral acquisition from (a) ETH Zurich and (b) the frequency sweep (Stanford), for different calculation methods: fifth order fit using 64 or 48 field probes (black), compressed fifth order fit (red), and conventional first order fit (blue). In both cases, better overall agreement was observed between the ground truth and compressed fifth order fit techniques, especially for second and third order terms.


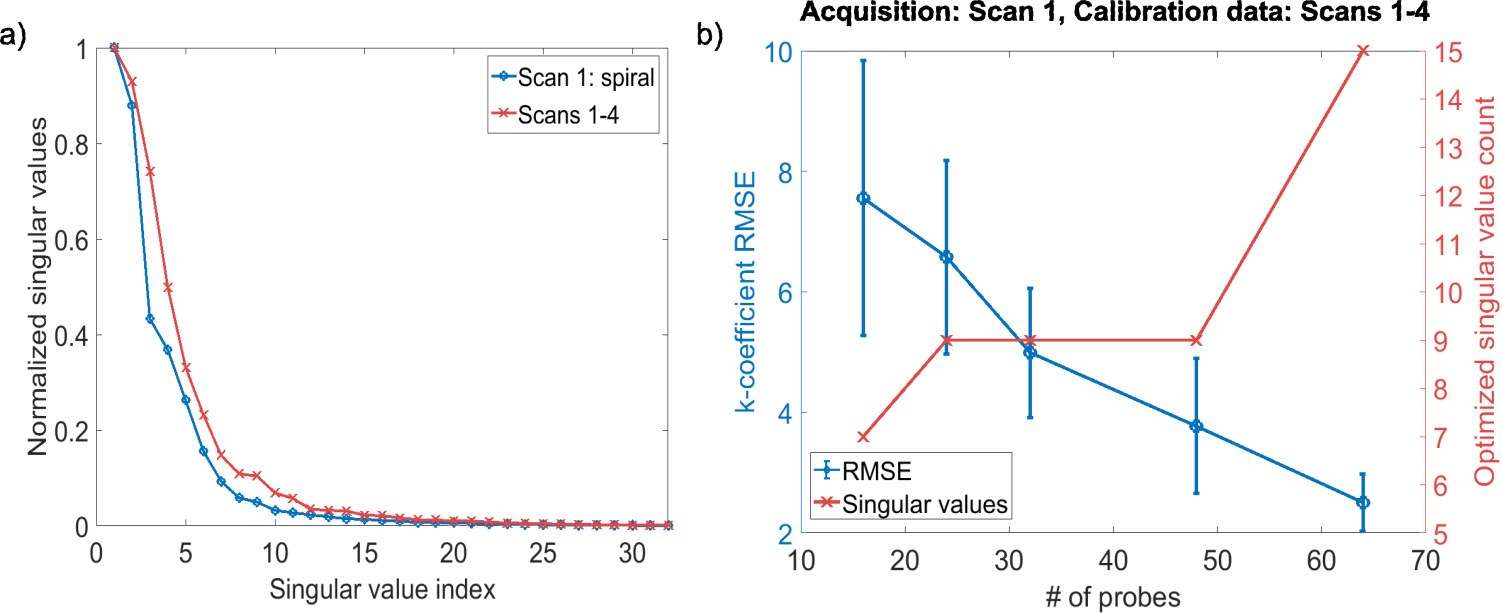


**Figure S5** (a) Singular value distributions for the following calibration datasets: 1.3-mm spiral acquisition “Scan 1” (blue) and for the combined calibration acquisitions “Scans 1-4” (red), with the combined calibration data exhibiting more prominent singular value weights. (b) illustrates mean k-coefficient RMSE (in blue) for the spiral acquisition (using Scans 1-4 combined calibration data) as a function of the following probe subset quantities provided during compressed fitting: 16, 24, 32, 48, 64. Probe subsets were determined by maximizing electrostatic repulsion from the 100-probe array. The red graph represents the respective number of singular values retained for compression following minimization of the RMSE: 7, 9, 9, 9, 15. With enough probes, the number of singular values that minimizes the RMSE increases, thereby also substantially reducing the RMSE. This suggests that the additional singular values possess valuable higher order modes. When reducing the number of preserved singular values, due to the constraints imposed by fewer probes, the error substantially increases, with the largest error observed for the 16-probe arrangement. The consistent minimization using 9 singular values when decreasing from 48 to 24 probes may be due to the importance of the 9^th^ singular value, as can be seen in the singular value plot. While preserving 9 singular values amounts to 13 total basis functions, which is still below the probe amount for the 16-probe case, fewer singular values (7) minimized the error likely because the poorer conditioning of the probing matrix when including more basis functions counteracts the benefits of including these significant singular values. Accordingly, compression performance proves to be limited for the Western setup when including differentiable calibration datasets.


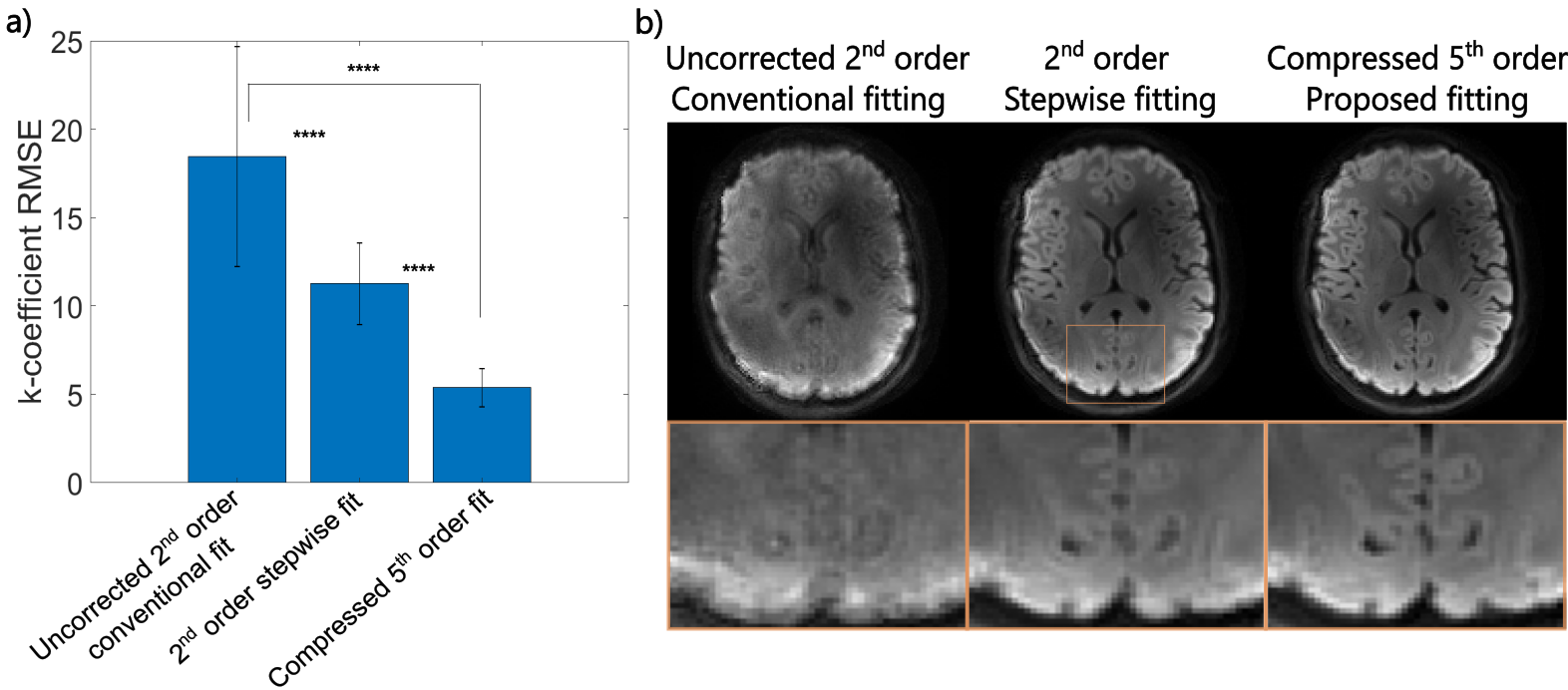


**Figure S6** Comparison of different proposed fitting techniques. (a) Quantitative k-coefficient RMSE analysis up-to-second order and (b) resulting qualitative mean DWI comparison informed by field dynamics computed conventionally with no form of fitting correction (left), using a previous fitting approach proposed by the authors (middle), and using the compressed basis function fitting approach (right). Improvements in both k-coefficient similarity and blurring reduction were observed with each successive iteration of fitting algorithm implemented.
